# Fe(II), Mn(II), and Zn(II) Binding to the C-Terminal Region of FeoB Protein: An Insight into the Coordination Chemistry and Specificity of the *Escherichia coli* Fe(II) Transporter

**DOI:** 10.1021/acs.inorgchem.3c02910

**Published:** 2023-11-01

**Authors:** Bartosz Orzel, Alessio Pelucelli, Malgorzata Ostrowska, Slawomir Potocki, Henryk Kozlowski, Massimiliano Peana, Elzbieta Gumienna-Kontecka

**Affiliations:** † Faculty of Chemistry, University of Wrocław, 50-383 Wrocław, Poland; ‡ Department of Chemical, Physical, Mathematical and Natural Sciences, University of Sassari, 07100 Sassari, Italy; || Department of Health Sciences, University of Opole, Katowicka 68, 45-060 Opole, Poland

## Abstract

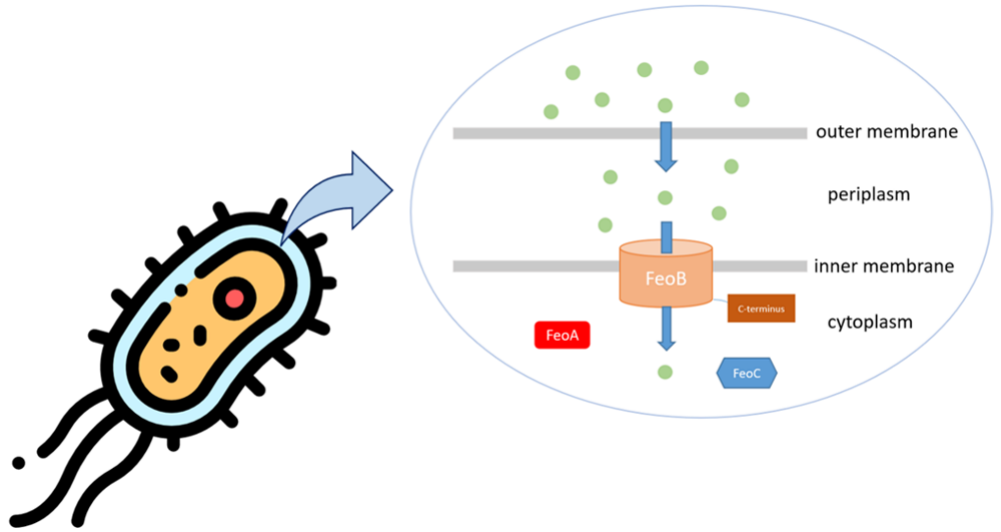

The interactions between two peptide ligands [Ac_763_CCAASTTGDCH_773_ (**P1**) and Ac_743_RRARSRVDIELLATRKSVSSCCAASTTGDCH_773_ (**P2**)] derived from the cytoplasmic C-terminal region of *Eschericha coli* FeoB protein and Fe(II), Mn(II), and Zn(II) ions were investigated. The Feo system is regarded as the most important bacterial Fe(II) acquisition system, being one of the key virulence factors, especially in anaerobic conditions. Located in the inner membrane of Gram-negative bacteria, FeoB protein transports Fe(II) from the periplasm to the cytoplasm. Despite its crucial role in bacterial pathogenicity, the mechanism in which the metal ion is trafficked through the membrane is not yet elucidated. In the gammaproteobacteria class, the cytoplasmic C-terminal part of FeoB contains conserved cysteine, histidine, and glutamic and aspartic acid residues, which could play a vital role in Fe(II) binding in the cytoplasm, receiving the metal ion from the transmembrane helices. In this work, we characterized the complexes formed between the whole cytosolic C-terminal sequence of *E. coli* FeoB (**P2**) and its key polycysteine region (**P1**) with Fe(II), Mn(II), and Zn(II) ions, exploring the specificity of the C-terminal region of FeoB. With the help of a variety of potentiometric, spectroscopic (electron paramagnetic resonance and NMR), and spectrometric (electrospray ionization mass spectrometry) techniques and molecular dynamics, we propose the metal-binding modes of the ligands, compare their affinities toward the metal ions, and discuss the possible physiological role of the C-terminal region of *E. coli* FeoB.

## Abstract 2

Fe(II) is the main source of iron in anaerobic conditions. An efficient iron uptake from the host environment is indispensable for the pathogenic bacteria during the infection. The Feo system, regarded as the most important bacterial Fe(II) uptake, consists usually of three proteins, of which transmembrane FeoB is directly involved in Fe(II) transport through the inner membrane. In this work, we characterize the coordination chemistry of the C-terminal part of FeoB.

## Introduction

Iron is an indispensable nutrient possibly for all living organisms, being involved in a plethora of crucial enzymatic processes. Due to its presence in heme and iron–sulfur clusters or bound to proteinogenic and other ligands, iron is a flexible cofactor for many enzymes involved in various life processes, such as cellular respiration, DNA synthesis and repair, and free-radical detoxification, just to name a few.^1−4^ During bacterial infection, the host organism limits the number of essential metal ions available for the pathogens.^5^ To grow and sustain pathogenicity, bacteria need to acquire indispensable iron from the host, making the ability to efficiently uptake iron from the environment the limiting factor for the survival of pathogenic bacteria.^6,7^ For this reason, bacteria have developed a variety of iron assimilation strategies. In aerobic conditions, iron is mostly present as Fe(III) ions, which bacteria uptake with the use of small organic chelators characterized by a very high affinity for Fe(III) ions, called siderophores.^8−10^ Most of the pathogenic bacteria can also acquire iron from the host’s proteins, such as lactoferrin and transferrin, by binding the proteins to the specific bacterial receptors and extracting iron from the protein’s structure.^11,12^ Heme iron can also be utilized by bacteria, either by direct heme binding to the receptors and transport to the cell or with the use of hemophores, which bind heme from the host environment and transport it back to the bacterial membrane.^13,14^ However, under anaerobic conditions, Fe(II) is the most prevalent form of iron. For bacteria occupying such environments, for example, predominantly anoxic gastrointestinal tracts of animals, the effective uptake of Fe(II) becomes one of the most important virulence factors.^15^ Despite the crucial role of Fe(II) in the pathogenicity, the detailed description of the mechanism of the bacterial uptake of Fe(II) is still relatively poorly understood, especially at the molecular level, compared to Fe(III) transport.

Divalent transition-metal ions are acquired by Gram-negative bacteria by proteins located in the inner membrane, facilitating ion transport from the periplasm to the cytoplasm.^16^ Transport through the outer membrane to the periplasm is thought to be most probable based on the free diffusion of metal ions through nonselective channels called porins; however, the presence of more selective outer membrane channels, for example, toward Mn(II) ions, is also proposed.^17−19^ Most of the Fe(II) inner membrane transporters are not specific toward a single metal ion and can transport a variety of them, e.g., Fe(II), Mn(II), Zn(II), Co(II), and Cu(II). Systems such as ZupT and YfeABCD are capable of transporting Fe(II), Mn(II), and Zn(II) ions.^20,21^ Apart from the above, Mn(II) and Fe(II) ions can also be acquired by the MntH transporter.^22^ Some bacteria, specifically members of the *Legionella* genus, express a Fe(II) transporter of higher specificity toward iron, called IroT. While shown in proteoliposome systems to transport a variety of divalent transition-metal ions, such as Mn(II), Zn(II), and Co(II), IroT is thought to serve solely as a Fe(II) transporter *in vivo*.^23−25^ In addition to all of the systems mentioned above, Feo appears to be both the most widespread and dedicated to Fe(II) transport.

**Figure 1:**
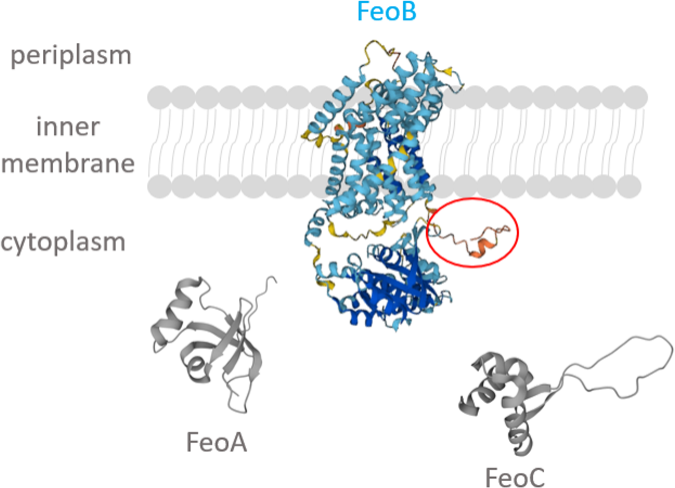
Schematic representation of the *E. coli* Feo system, which consists of cytoplasmic FeoA and FeoC proteins and a transmembrane FeoB protein. The C-terminal part of FeoB is marked with the red circle. The scheme does not show the true relationships between the size of the proteins or their factual orientation. The protein structures were generated using UniProt and AlphaFold (UniProt entries: FeoA-P0AEL3, FeoB-P33650, and FeoC-P0AEL3).^33^

The Feo system is regarded as the most important Fe(II) transporter in bacteria, crucial for iron acquisition under anaerobic conditions.^16,26,27^ Mutations or deletions in genes encoding Feo system proteins result in serious impairment of Fe(II) ion acquisition and virulence in some bacterial species, like *Streptococcus suis*, *Campylobacter jejuni*, and *Helicobacter pylori*.^28−30^ The Feo system was first described in *Escherichia coli* in 1987.^31^ The *E. coli* Feo system is tripartite (Figure 1), with cytoplasmic FeoA and FeoC proteins, and a transmembrane FeoB protein, encoded in *feoABC* operon.^32^ Other operon organizations can be *feoB*, *feoAB* fusion, and *feoAB*, which is actually the most prevalent.^16,27^ Therefore, while FeoA and FeoC can be a part of the Feo system, transmembrane FeoB is an indispensable component of the Feo.

FeoA and FeoC are small cytosolic proteins with a mass of about 8 kDa. It has been suggested that FeoA might interact with FeoB, while FeoC could act as a transcription factor; however, the exact roles of these proteins are yet to be elucidated.^26,27^

**Figure 2:**
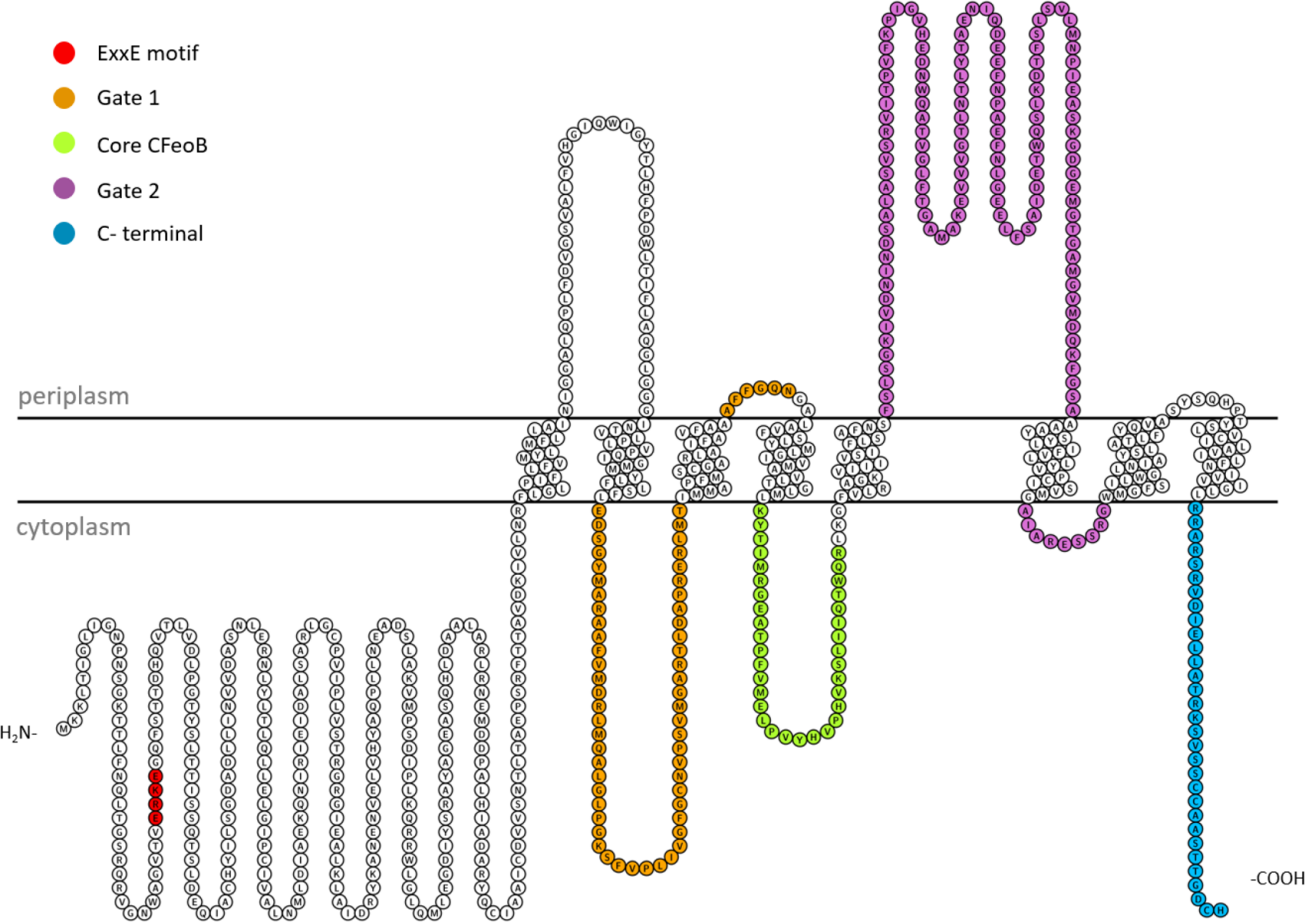
Predicted topology diagram of *E. coli* FeoB, with the periplasmic and cytoplasmic parts of putative Fe(II) binding regions marked in colors. Topology prediction carried out by the DeepTMHMM server, visualized with the use of the Protter tool.^39,40^

Because FeoB is an indispensable component of the Feo system and transports the metal ion through the membrane, it has been extensively studied; however, to date, there is a lack of consensus about the mechanism of Fe(II) transport by FeoB. It is a transmembrane protein consisting of 773 amino acids in *E. coli* and possibly 8 transmembrane helices. The *E. coli* FeoB structure can be divided into a N-terminal NFeoB domain, found in the cytoplasm, a transmembrane domain, and a cytoplasmic C-terminal part. The NFeoB domain contains high sequence homology with GTP-binding proteins; thus, it was suggested that FeoB transports Fe(II) in an active manner, utilizing the energy from GTP hydrolysis in the N-terminal domain.^26,27,34^ It was also shown that the GTP-binding domain is essential for Fe(II) uptake.^34^ As yet, there is no consensus on whether FeoB could work in an active manner because most of the obtained FeoB’s GTP-hydrolysis rates are too low to drive efficient iron transport through the membrane.^35,36^ Furthermore, there is no consensus on which amino acid residues are involved in Fe(II) binding and transport. There are a few proposed putative metal binding sites in the protein sequence, for example, the Gate 1 and Gate 2 motifs, reminiscent of the yeast iron permease Ftr1p found in the periplasmic, cytoplasmic, and transmembrane parts of the protein, the Core CFeoB region found in the fourth transmembrane helix and the cytoplasmic insertion between the fourth and fifth helices, and the ExxE motif located in the cytoplasmic GTP-binding domain (Figure 2).^16,26,37,38^

**Figure 3:**
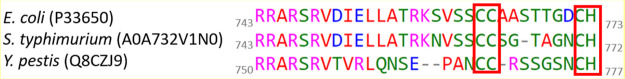
Comparison of the FeoB cytoplasmic C-terminal sequences from various bacteria belonging to the gammaproteobacteria class. The conserved cysteine and histidine residues are marked by red rectangles. UniProt entry codes are given in brackets. Alignment carried out with Clustal Omega.^41^

All of these putative binding sites contain conserved residues believed to be able to bind Fe(II) with good affinity. Another potential Fe(II) binding place recognized in the literature could be the C-terminal fragment of the protein.^16,37^ In the gammaproteobacteria class (e.g., *E. coli*, *Y. pestis*, and *S. typhimurium*), the cytoplasmic C-terminal fragment of FeoB protein is rich in strongly conserved cysteine residues, with histidine and glutamic and aspartic acid residues also present, which could act as metal binding ligands in the cytoplasm after the transfer of the metal ion through the membrane (Figure 3). While Feo is generally considered to be Fe(II)-specific, many Fe(II) bacterial acquisition systems can also transport Mn(II) and Zn(II) ions, as shown above.

According to Pearson’s theory of hard and soft acids and bases, the carboxy-terminal FeoB sequence possesses efficient complexing ligands for both hard Mn(II) ion (for example, oxygen atoms of aspartic and glutamic acid residues) and more borderline Fe(II) and Zn(II) (e.g., sulfur atoms of cysteine residues).^42,43^ Additionally, there are proteins in which manganese can also be coordinated by cysteine’s sulfur, as well as histidine’s nitrogen atoms, for example, in the case of calprotectin.^44−46^ Therefore, we decided to examine the metal-binding properties of carboxyl-terminal *E. coli* K12 FeoB fragments. Drug-resistant *E. coli* is becoming a global concern because pathogenic strains of this bacteria were the leading cause of death linked to antimicrobial resistance in 2019.^47^ It is estimated that drug-resistant *E. coli* will be responsible for 3 million deaths each year by 2050.^48^ An alternative way of treating bacterial infections could be inhibition of the Fe(II) transition, resulting in iron starvation of the bacteria. This could be especially effective for pathogenic bacteria occupying the anoxic environments, for which effective Fe(II) transport is one of the most important virulence factors.^15^ Furthermore, mutations or deletions of genes encoding the Feo system can lead to reduced virulence.^28−30^ Coordination chemistry studies of Fe(II) with bacterial transporters are necessary to determine the metal-binding sites of the transporters and thus elucidate the mechanism of Fe(II) transport; however, they are significantly lacking in the literature, most likely because of the difficulties of working with an oxidation-prone Fe(II) ion and maintaining the anaerobic conditions throughout the experiments. Aware of these important issues, we decided to examine the metal- binding properties of the C-terminal part of *E. coli* K12 FeoB.

In order to do that, we chose two peptide sequences from *E. coli* K12 strain FeoB protein that differ in length: peptide 1 [Ac_763_CCAASTTGDCH_773_ (**P1**)] is the shorter fragment that we selected to study in detail the importance of the aspartic D751 (D_8_ in this work) and glutamic E753 (E_10_ in this work) acid residues for metal binding, present in peptide 2 [Ac_743_RRARSRVDIELLATRKSVSSCCAASTTGDCH_773_ (**P2**)]. **P2** is the complete cytoplasmic part of C-terminal FeoB. The N-terminal amino acids were acetylated to resemble the native protein. In this work, we examined the thermodynamics of Fe(II), Zn(II), and Mn(II) complexes with the aforementioned peptides; i.e., we determined the protonation constants of peptides, the stability constants of complexes, their stoichiometry, and the proposed coordination modes, all with the use of potentiometry, mass spectrometry, and NMR and electron paramagnetic resonance (EPR) spectroscopies. Overall, the scope of this work was to determine the specificity of Fe(II), Zn(II), and Mn(II) ion binding by the carboxy-terminal fragment of *E. coli* FeoB to understand whether it could bind Mn(II) and Zn(II) in addition to Fe(II).

## Experimental Section

### Materials

Both ligands (**P1** and **P2**) were purchased from KareBay Biochem and were of 98% purity. The identity of the peptides was evaluated based on mass spectrometry. The purity was checked based on potentiometric titrations using the Gran method.^49^ Carbonate-free 0.1 M Titripur sodium hydroxide was purchased from Sigma-Aldrich and standardized by titrations with potassium hydrogen phthalate (Sigma-Aldrich). Zn(II) and Mn(II) ion solutions were made from corresponding perchlorate salts (Sigma-Aldrich) and standardized using two different methods: inductively coupled plasma optical emission spectrometry (ICP-OES) and complexometric titrations with standardized ethylenediaminetetraacetic acid disodium salt (Na_2_H_2_EDTA) and murexide. A Fe(II) ion solution was prepared right before the experiments from ammonium iron(II) sulfate (Sigma-Aldrich) and standardized using 1,10-phenanthroline (Sigma-Aldrich) colorimetric assay under an inert atmosphere. The ionic strength was adjusted to *I* = 0.1 M using sodium perchlorate (Sigma-Aldrich). Ligand samples also included 4 mM perchloric acid (J. T. Baker). All samples were prepared using double distilled water. Due to the oxidation sensitivity of Fe(II), all samples for Fe(II) experiments were prepared in an argon atmosphere inside a glovebox using deoxygenated solvents. For Fe(II) experiments, we did not observe the presence of Fe(III). In mass spectrometry spectra, no peaks that could be assigned to Fe(III) complexes were present. During the potentiometric titrations, the sample solution was transparent and colorless throughout the entire pH range. After finished titrations, opening the potentiometric vessel and exposing the solution to air resulted in the rapid formation of a yellow color, a consequence of Fe(II)-to-Fe(III) oxidation (Figure S1). Samples after the NMR experiments were treated with thiocyanate anions in an anaerobic atmosphere; however, no formation of the red Fe(III) complex was observed. All of the Fe(II) samples prepared for experiments were colorless. Thus, we firmly believe that our procedure of Fe(II) sample preparation was very careful, and no iron oxidation took place during the experiments.

### Electrospray Ionization Mass Spectrometry (ESI-MS)

ESI-MS studies were conducted using a Shimadzu LCMS-9050-QTOF mass spectrometer. Positive-ion mode spectra of samples containing a 0.1 mM ligand concentration and a 1:1 or 1:2 (metal/ligand) molar ratio were recorded. Samples were prepared in a 50:50 (w/w) methanol/water solvent at pH 7.4. Spectra were recorded in the *m*/*z* 200–2000 range. The injection volume was 1 μL. Conditions: nebulizing gas, nitrogen; nebulizing gas flow, 3.0 L/min; drying gas flow, 10 L/min; heating gas flow, 10 L/min; interface temperature, 300 °C; desolvation line temperature, 400 °C; detector voltage, −2.02 kV; interface voltage, 4.0 kV; collision gas, argon; mobile phase, MeCN + 0.1% HCOOH. The obtained signals had a mass accuracy error in the range of 1 ppm. All used solvents were of liquid chromatography–mass spectrometry grade. The obtained data were analyzed with *LabSolutions* software (Shimadzu, Kyoto, Japan).

### Potentiometric Titrations

A Metrohm Titrando 905 titrator connected to a Dosino 800 dosing system was used for potentiometric titrations. The pH of the sample solutions was measured by a pH electrode, InLab Semi-Micro (Mettler-Toledo). The thermostabilized cell glass was equipped with a microburet delivery tube, a magnetic stirrer, and an inlet–outlet tube for argon. The stability constants of the proton and Fe(II), Zn(II), and Mn(II) complexes with ligands were calculated using titration curves from pH 2 to 11 at a temperature of 298 K, by *SUPERQUAD* and *HYPERQUAD 2008* software.^50,51^ Sample solutions contained concentrations of 0.5 mM ligand, 4 mM perchloric acid, and 0.1 M sodium perchlorate (ionic strength). The exact concentrations of the ligand solutions were determined by the Gran method. In metal complex titrations, a molar ratio of 1:1.1 or 1:2 (metal/ligand) was used. All titrations were performed under an argon atmosphere, using carbonate-free, standardized sodium hydroxide as the base. The electrode was calibrated every day for the hydrogen ion concentration by titrating 2 mL of 4 mM perchloric acid with sodium hydroxide. Standard deviations were calculated by *HYPERQUAD 2008* and are referred to as random errors only. The competition and speciation diagrams were created using *HYSS* software.^52^ Fe(II), Zn(II), and Mn(II) hydrolysis constants were taken into account for calculations of the stability constants of complexes. The hydrolysis constants for zero ionic strength were taken from *The Hydrolysis of Metal Cations* by Brown and Ekberg and calculated to 0.1 M ionic strength with the formula proposed by Baes and Mesmer in *The Hydrolysis of Cations*.^53,54^ The metal hydrolysis constants are collected in Table S1.

### NMR Spectroscopy

NMR experiments were performed using a Bruker Ascend 400 MHz spectrometer equipped with 5 mm automated tuning and a matching broadband probe (BBFO) with *z* gradients. Samples used for NMR experiments were in the range 0.4–1.0 mM and dissolved in 90:10 (v/v) H_2_O/D_2_O. All NMR experiments were performed at 298 K in 5 mm NMR tubes. The 2D ^1^H–^13^C heteronuclear correlation (HSQC) spectra were acquired using a phase-sensitive sequence employing Echo-Antiecho-TPPI gradient selection with a heteronuclear coupling constant *J*_XH_ = 145 Hz and shaped pulses for all 180° pulses on the f2 channel with decoupling during acquisition. Sensitivity improvement and gradients in back-inept were also used. Relaxation delays of 2 s and 90° pulses of about 10 μs were applied for all experiments. Solvent suppression was achieved by using excitation sculpting with gradients. The spin-lock mixing time of the ^1^H–^1^H total correlation spectroscopy (TOCSY) experiment was obtained with MLEV17. ^1^H–^1^H TOCSY experiments were performed using a mixing time of 60 ms. ^1^H–^1^H rotating frame Overhause effect spectroscopy (ROESY) spectra were acquired with spin-lock pulse durations in the range of 200–250 ms. The assignments of ^1^H and ^13^C were made by a combination of mono- and bidimensional and multinuclear NMR techniques: ^1^H–^1^H TOCSY, ^1^H–^13^C HSQC, and ^1^H–^1^H ROESY, at different pH values. To avoid severe broadening of the signals, because of the paramagnetic character of Mn(II) and Fe(II), the NMR experiments were performed with the subsequent addition of a substoichiometric amount of metal ion to the ligand solution. All NMR data were processed using *TopSpin* (Bruker Instruments) software and analyzed using the *Sparky 3.11* and *MestReNova 6.0.2* (Mestrelab Research S.L.) programs.

### EPR Spectroscopy

EPR spectra were recorded using a Bruker ELEXSYS E500 CW-EPR spectrometer equipped with an NMR teslameter (ER 036TM) and a frequency counter (E 41 FC) at X-band frequency and 77 K and room temperature. The peptide concentration was 1 mM, and the metal/ligand molar ratio was 1:1.1. The solution for EPR experiments was prepared by using ethylene glycol (5%) as a cryoprotectant. EPR parameters were obtained by using the Bruker WinEPR SimFonia program and Doublet new (EPR OF; *S* = ^1^/_2_) program by A. Ozarowski (National High Field Magnetic Laboratory, University of Florida, Gainesville, FL).

### UV–Vis Spectroscopy

The absorption spectra were recorded under an inert atmosphere using Jasco V-730 UV–vis spectrophotometer in the 350–650 nm range, using a quartz cuvette with a 0.1 cm optical path, a scanning speed of 400 nm/m, a data pitch of 0.5 nm, and a number of accumulations of 1. The colorimetric Fe(II) concentration determination utilized the formation of a 1:3 metal/ligand complex of Fe(II) with 1,10-phenanthroline, with λ_max_ = 510 nm. First, the calibration curve was prepared for Fe(II) ion concentrations in the range of 0.1–1.1 mM, and a linear function correlating the absorption of the solution with a Fe(II) concentration was obtained. Then, the calibration curve was used to determine the concentration of the freshly prepared Fe(II) stock solution, by measuring the absorption at 510 nm of the three samples made from the stock solution and taking the average of the concentration calculated for each sample. The ratio of Fe(II) to 1,10-phenanthroline was 1:5 to ensure complete complexation of the metal ion.

### Molecular Dynamics Measurements

Extended peptide conformers were generated in *Avogadro*.^55^ Energy minimization was achieved using a restricted Hartree–Fock self-consistent field, and calculation was performed using Pulay DIIS + Geometric Direct Minimization with basis set 3-21G(*) using *Gaussian 16*. Subsequent calculations were carried out using *NAMD*(56) and *VMD 1.9.3* software.^57^ Structures were parametrized in CHARMM-GUI^58^ using the CHARMM36m force field. A rectangular waterbox was used to simulate the solvent behavior, sized as the protein size plus 5 Å more on each side of the waterbox, and completed with 0.05 M KCl to balance the charge of the deprotonated amino acids. Additional ions were placed by using the Monte Carlo method. The simulations were carried out at a temperature of 298 K. Each simulation was executed for 1 μs (following an initial minimization), with structures calculated every 10 ps and written to a trajectory file. For the 1 μs simulation, 50000 time steps were recorded (2 fs = step size). Following the calculations, root-mean-square deviation (RMSD) data plots generated relative to the extended, minimized initial structure were generated from the trajectory. The structures shown in the RMSD trajectories were extracted using *VMD 1.9.3* and visualized with *ChimeraX 1.3*.^59^

## Results and Discussion

Fe(II), Zn(II), and Mn(II) complexes with **P1** and **P2** have been investigated by a combination of potentiometric, spectroscopic (NMR and EPR), and spectrometric (ESI-MS) techniques. Potentiometric titrations allowed us to calculate the protonation constants of ligands and the stability constants of complexes as well as to draw the speciation and competition diagrams in the pH range of 2–11. ESI-MS measurements indicated the stoichiometry of the formed complexes, while spectroscopic experiments confirmed the metal-binding residues and provided insight into the geometry of the metal complexes.

**P1** and **P2** ligands contain the following residues able to deprotonate in the pH range of 2–11: aspartic and glutamic acid side-chain carboxyl groups, an imidazole ring nitrogen of histidine, the thiol group of cysteine, and a carboxy-terminal group. **P1** consists of 11 amino acids, while **P2** consists of 31 amino acids, containing the whole cytosolic carboxy-terminal fragment of *Ec*FeoB.

Under the studied pH conditions, **P1** behaves like an H_6_L acid (Figure S2a). The first two dissociation constants are ascribed to the deprotonation of carboxylic groups: first from the C-terminal group (p*K*_a_ = 3.37) and second from the aspartic acid residue (D_29_, p*K*_a_ = 4.19). The next constant comes from the histidine’s (H_31_) imidazole nitrogen deprotonation (p*K*_a_ = 6.96), and the last three arise from the cysteine residues’ (C_21_, C_22_, and C_30_) deprotonation (p*K*_a_ values in the range from 8.15 to 9.81).

**P2**, being 20 amino acids longer than **P1**, has only two more groups able to deprotonate in the tested pH range. These are the carboxylic groups of another aspartic acid (D_8_) and a glutamic acid residue (E_10_). Thus, **P2** behaves like an H_8_L acid, with corresponding p*K*_a_ values similar to those of **P1** (Figure S2b). The deprotonation starts from the C-terminal group (p*K*_a_ = 2.15), with two aspartic acids (D_8_ and D_29_; p*K*_a_ values of 3.12 and 3.74; however, it is not possible to assign these to specific aspartic acids by potentiometry), glutamic acid (E_8_; p*K*_a_ = 4.38), and histidine (H_31_; p*K*_a_ = 6.73) deprotonating next. The p*K*_a_ values of the three cysteine residues (C_21_, C_22_, and C_30_) in **P2** are in the range of 8.15–10.11. It is important to highlight that **P2** contains a lysine (K_16_) residue, which usually exhibits p*K*_a_ values between 10 and 10.50. However, we could not determine the p*K*_a_ value for the lysine residue in **P2**. We believe this is a consequence of the high p*K*_a_ value of the third cysteine residue (which is 10.11), which shifts the lysine deprotonation well above pH 11, which is the upper limit of the experimental pH range. Such behavior has already been observed by us in other systems.^60^ The ligand protonation constants are listed in Table 1. Speciations for **P1** and **P2** are presented in Figure S2.

**Table 1:** Protonation Constants (log β) and p*K*_a_ Values of Peptides **P1** and **P2**^a^

peptide	species	log β^b^	p*K*_a_^c^	deprotonating residue
**P1**	[H_6_L]^+^	41.40(4)	3.37	C_terminal_
	[H_5_L]	38.03(4)	4.19	Asp
	[H_4_L]^−^	33.84(3)	6.96	His
	[H_3_L]^2–^	26.88(3)	8.15	Cys
	[H_2_L]^3–^	18.73(2)	8.92	Cys
	[HL]^4–^	9.81(2)	9.81	Cys
**P2**	[H_8_L]^7+^	47.41(2)	2.15	C_terminal_
	[H_7_L]^6+^	45.26(2)	3.12	Asp
	[H_6_L]^5+^	42.14(2)	3.74	Asp
	[H_5_L]^4+^	38.40(2)	4.38	Glu
	[H_4_L]^3+^	34.02(2)	6.73	His
	[H_3_L]^2+^	27.29(1)	8.15	Cys
	[H_2_L]^+^	19.14(1)	9.03	Cys
	[HL]	10.11(1)	10.11	Cys

a *T* = 298 K; *I* = 0.1 M NaClO_4_; standard deviations on the last digit given in parentheses.

b Overall stability constants (β) expressed by the equation β(H_*n*_L) = [H_*n*_L]/[L][H^+^]^*n*^.

c Acid dissociation constants (p*K*_a_) expressed as p*K*_a_ = log β(H_*n*_L) – log β(H_*n*–1_L).

### Metal Complex Stoichiometry

**Figure 4:**
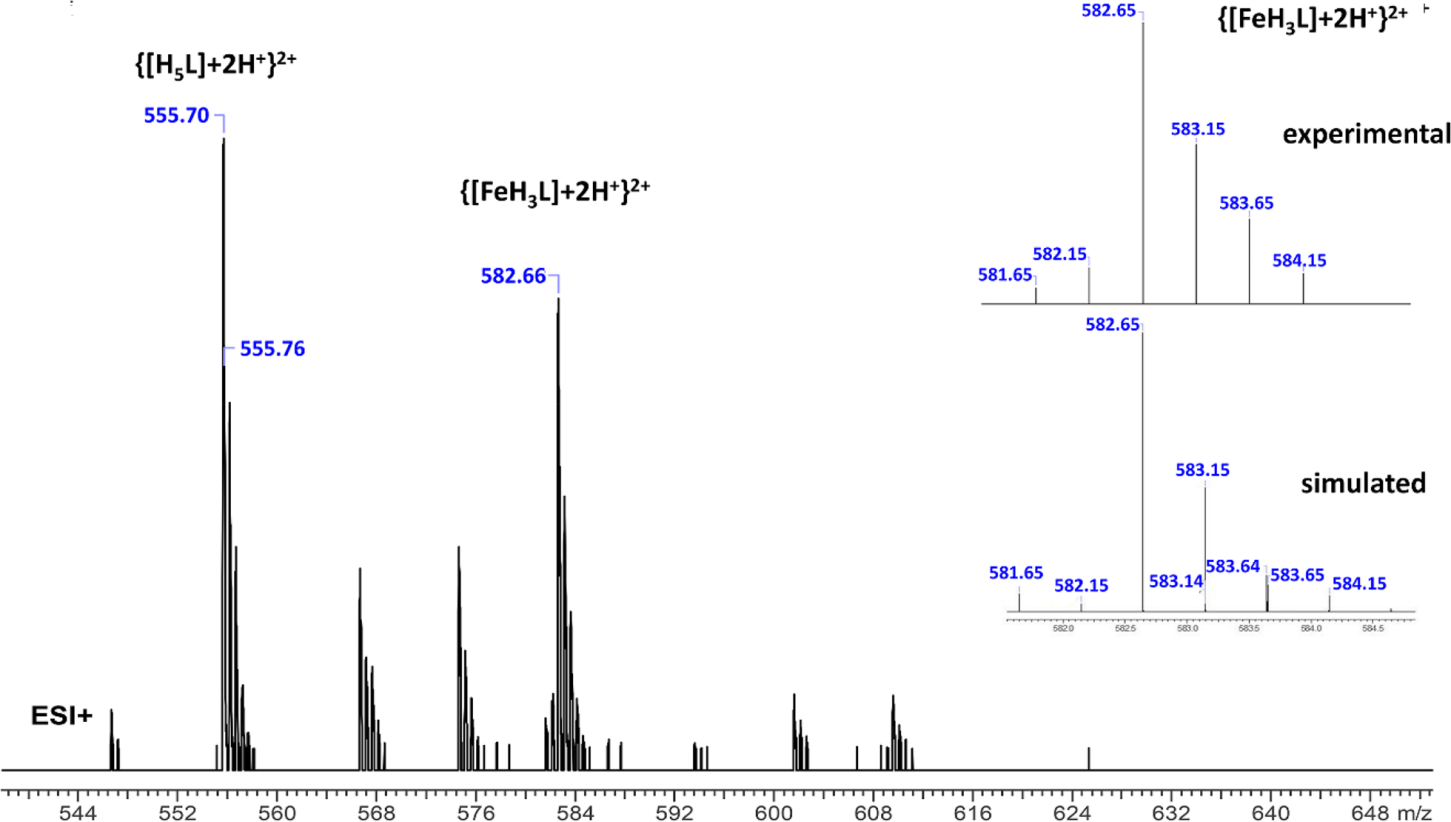
ESI-MS spectrum of the Fe(II)/**P1** system at pH 7.4 and M:L = 1:1. The experimental and simulated isotopic distribution spectra of the peak at *m*/*z* 582.66 are shown in the upper right corner.

The stoichiometry of complexes formed by Fe(II), Zn(II), and Mn(II) with **P1** and **P2** was first determined by ESI-MS experiments, which revealed only the formation of mononuclear 1:1 (M/L) complexes. The mass spectrum for the Fe(II)/**P1** system is shown in Figure 4 as a representative example. The correct peak assignment was confirmed by comparing the peak’s simulated and experimental isotopic distributions. A comparison of the experimental and simulated *m*/*z* values of the signals of ionized metal complexes and ligands present in the spectra of each metal/peptide system is presented in Table S2.

#### Iron Complexes

**Figure 5:**
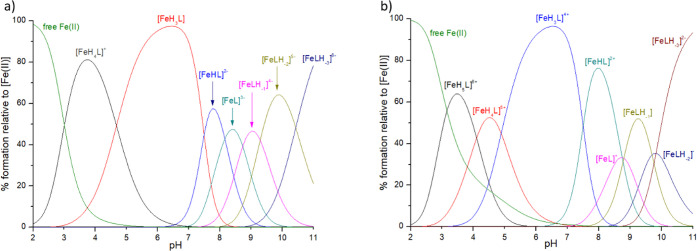
Distribution diagrams of complexes formed between Fe(II) and ligands: (a) ligand **P1**; (b) ligand **P2**. Species distribution calculated for NMR experimental conditions: [Fe(II)]_tot_ = 0.26 mM; Fe(II):L = 1:3.

For the Fe(II)/**P1** system, the first form seen in the potentiometry is [FeH_4_L]^+^, which is present in the solution from the start of the titration (Figure 5a). This form probably contains a C-terminal carboxyl group and aspartic acid (D_29_) in the deprotonated state. Most probably at least one COO^–^ is involved in the metal binding, with D_29_ involvement proven by the NMR spectra at pH 6.3. The next form, [FeH_3_L], is possibly related to the histidine residue (H_31_) deprotonation and dominates in the solution throughout a wide pH range, from about 4.7 to 7.5.

**Figure 6:**
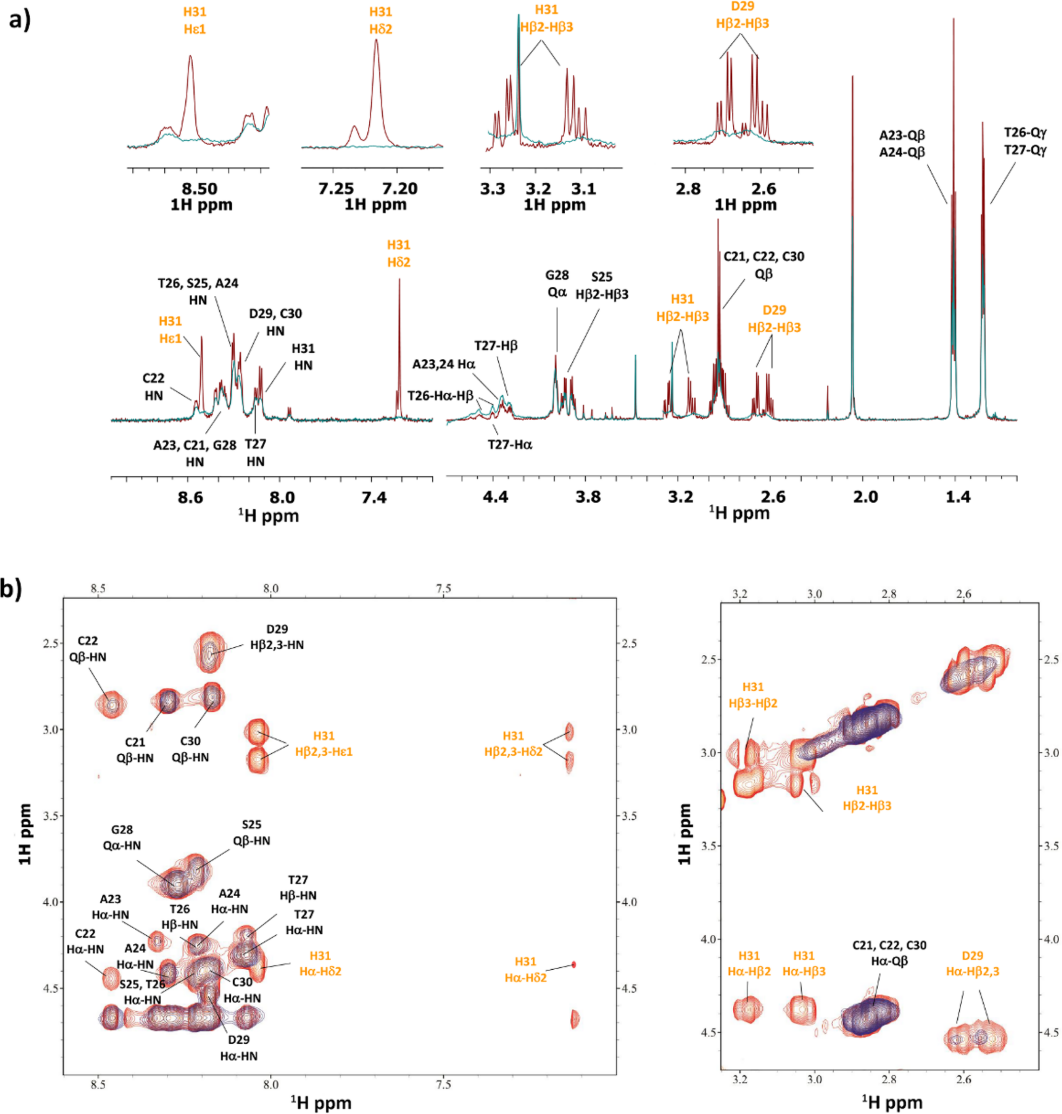
(a) Comparison of ^1^H spectra and (b) selection of ^1^H–^1^H TOCSY spectra for the free peptide **P1** (red) and Fe(II)/**P1** system (blue) at 1:3 molar ratio and pH 6.3.

This form contains over 90% Fe(II) in the solution at pH 6.5. The p*K*_a_ value of 4.68 is significantly decreased compared to the p*K*_a_ value of 6.96 for this residue in the free ligand, suggesting the participation of a histidine side chain in the coordination. The {COO^–^, N_im_} binding mode is confirmed by the NMR spectra recorded at pH 6.3, in which the perturbation of H_31_ and D_29_ can be seen, indicating the involvement of these residues in metal coordination (Figure 6). The involvement of terminal COO^–^ of H_31_, as depicted in the proposed structural model in Figure S3, cannot be excluded.

**Figure 7:**
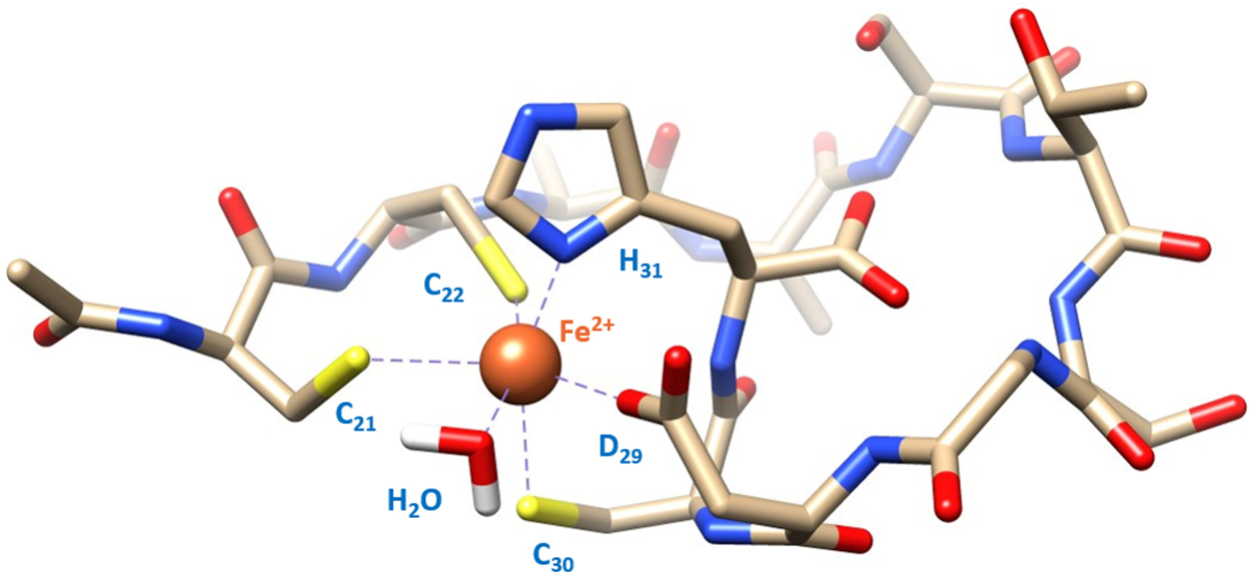
Structural model of the [FeL]^3–^ species in a {COO^–^, N_im_, 3 S^–^, H_2_O} coordination mode.

Most probably, the deprotonation of two cysteine residues leads to the formation of [FeHL]^2–^. The [FeH_2_L]^−^ form could not be detected by potentiometry, probably being just a transient form whose concentration in the solution is low. The last cysteine residue deprotonation corresponds to [FeL]^3–^ formation. The lowered p*K*_a_ value of this cysteine in the complex (p*K*_a_ = 8.16) compared to the ligand (p*K*_a_ = 9.81) suggests its involvement in metal binding. This is consistent with the NMR spectra recorded at pH 8.16. At this pH, there is a mixture of complex forms in the solution, with [FeL]^3–^ being the dominating one. In the NMR spectra, the disappearance of signals related to C_21_, C_22_, D_29_, C_30_, and H_31_ can be observed, confirming all of the cysteine residues taking part in the Fe(II) binding in the {COO^–^, N_im_, 3 S^–^} mode (Figures 7 and S4a). The last three forms, starting from [FeLH_–1_]^4–^, most probably result from the deprotonation of water molecules or amide groups of the peptide. The NMR spectra recorded at pH 9.45, at which all three forms, [FeLH_–1_]^4–^, [FeLH_–2_]^5–^, and [FeLH_–3_]^6–^, are present in the solution, show less perturbation on D_29_ and H_31_. This could indicate a decreasing involvement of these residues in metal binding and could mean that other atoms enter the coordination sphere (Figure S4b). It could be the nitrogen atoms from the three amide groups of the peptide bonds starting to coordinate with the Fe(II) ion together with the three cysteine residues, resulting in a hexacoordinated Fe(II) complex, with nitrogen and sulfur atoms being the only ligands in the {3 S^–^, 3 N^–^} mode. The possibility of Fe(II) binding by amides has already been documented in the literature, not only for peptides but also for macrocycles and other ligands.^61−67^ Moreover, taking into consideration the borderline acid character of Fe(II), the displacement of oxygen ligands by nitrogen donors would most probably result in more stable complexes. With scarce literature regarding the peptide complexes of Fe(II), there are almost no data on the p*K*_a_ values of amide nitrogen in such systems; however, values obtained by us are consistent with those proposed in recent work on Fe(II)/peptide systems,^67^ suggesting that indeed amides likely enter the Fe(II) coordination sphere under alkaline pH conditions. Still, one cannot exclude an alternative interpretation of these three p*K*_a_ values, corresponding to the dissociation of water molecules bound to the Fe(II) ion. A proposed structure of [FeLH_–2_]^5–^ species with two amide groups and one water molecule coordinated to the metal atom is shown in Figure S5. The stability constants of the complexes in the Fe(II)/**P1** system, as well as Fe(II)/**P2**, are collected in Table 2.

**Table 2:** Stability Constants (log β) and p*K*_a_ Values of the Fe(II)/Peptide Systems^a^

peptide	species	log β^b^	p*K*_a_^c^	deprotonating residue
**P1**	[FeH_4_L]^+^	38.76(2)		
	[FeH_3_L]	34.08(4)	4.68	His
	[FeHL]^2–^	19.10(5)		Cys, Cys
	[FeL]^3–^	10.94(5)	8.16	Cys
	[FeLH_–1_]^4–^	2.21(6)	8.73	N_amide_/O_water_
	[FeLH_–2_]^5–^	–7.08(5)	9.29	N_amide_/O_water_
	[FeLH_–3_]^6–^	–17.52(5)	10.44	N_amide_/O_water_
**P2**	[FeH_5_L]^6+^	42.75(2)		
	[FeH_4_L]^5+^	38.65(4)	4.13	Glu
	[FeH_3_L]^4+^	33.75(5)	4.90	His
	[FeHL]^2+^	18.76(5)		Cys, Cys
	[FeL]^+^	10.01(7)	8.75	Cys
	[FeLH_–1_]	1.21(6)	8.80	N_amide_/O_water_
	[FeLH_–2_]^−^	–8.48(7)	9.69	N_amide_/O_water_
	[FeLH_–3_]^2–^	–18.31(6)	9.83	N_amide_/O_water_/Lys

a *T* = 298 K; *I* = 0.1 M NaClO_4_; standard deviations given in parentheses.

b Overall stability constants (β) expressed by the equation β([FeH_*n*_L](^*n*+2^)^+^) = [[FeH_*n*_L]^(*n*+2)+^]/[Fe(II)][[L]^*n*+^][H^+^]^*n*^.

c Acid dissociation constants (p*K*_a_) expressed as p*K*_a_ = log β([FeH_*n*_L]^(*n*+2)+^) – log β([FeH_*n*–1_L]^(*n*+1)+^).

**Figure 8:**
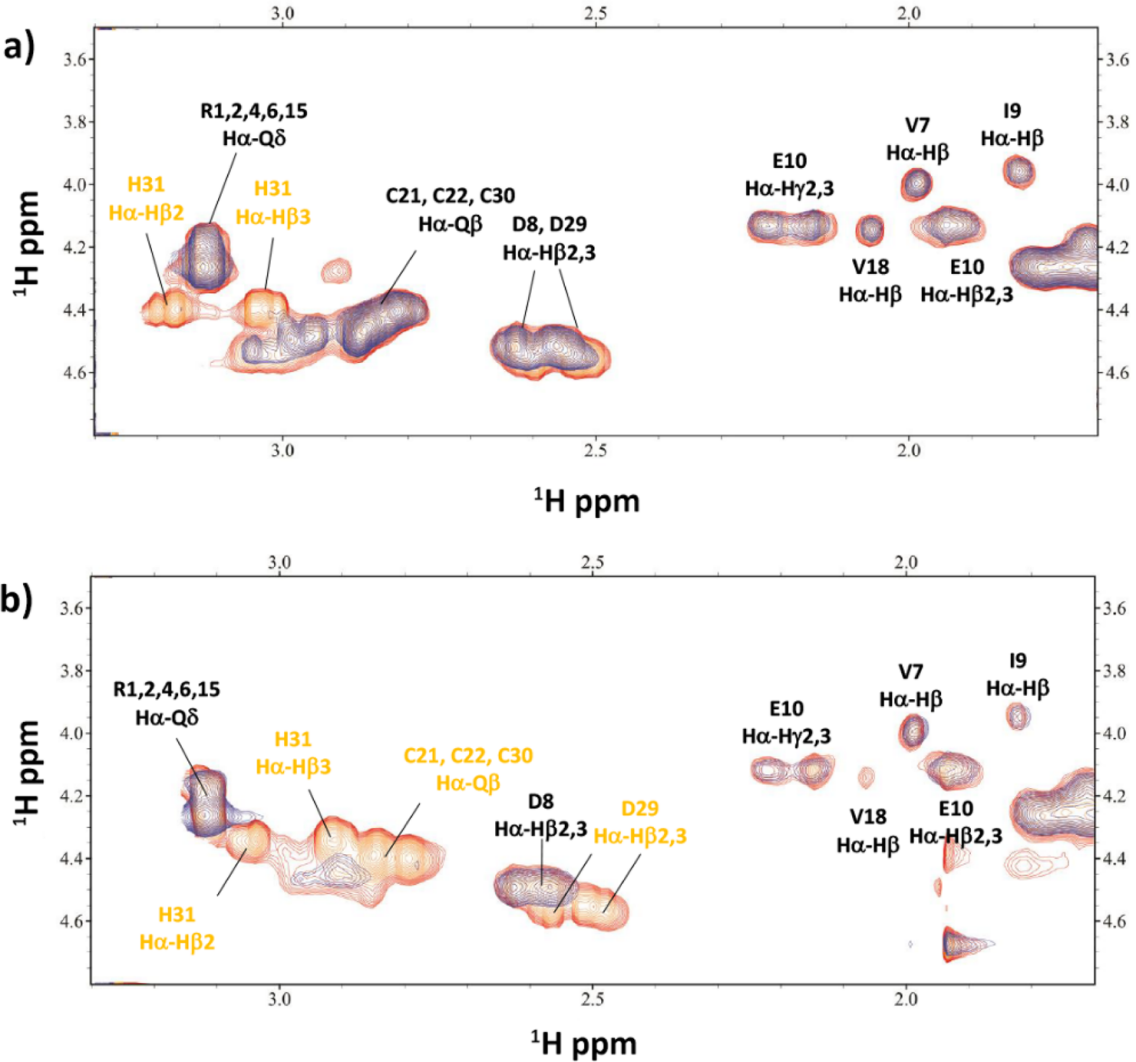
Comparison of a selection of ^1^H–^1^H TOCSY spectra for the free peptide **P2** (red) and Fe(II)/**P2** system (blue) at a 1:3 molar ratio and pH 6.2 (a) and 8.47 (b).

In the Fe(II)/**P2** system, we observe a variety of forms throughout the studied pH range (Figure 5b). The first form calculated in potentiometric models is [FeH_5_L]^6+^, in which most probably the carboxyl C-terminal group and two aspartic acid residues are deprotonated. From potentiometric results, it is not possible to determine whether these groups take part in iron binding; however, the NMR spectra recorded at pH 6.2 and 8.47 prove that indeed D_29_ is involved in metal binding. This form is present in the solution from the start of the titration. The deprotonation of the glutamic acid (E_10_) residue possibly leads to the [FeH_4_L]^5+^ formation (the p*K*_a_ value of 4.13 is almost the same as that in the free ligand), suggesting that this residue does not participate in metal binding. From a pH of about 4.90, the [FeH_3_L]^4+^ form dominates in the solution, all the way up to a pH of about 7.5, resembling the [FeH_3_L] form observed for **P1**. Both of them are probably the first forms in the solution to contain the deprotonated histidine (H_31_), with its p*K*_a_ value of 4.90 significantly lower than that in the free ligand, suggesting the imidazole nitrogen involvement in metal binding. This is indeed confirmed by the NMR spectra acquired at a pH of 6.2, in which perturbation of the signals related to H_31_ can clearly be seen (Figures 8a and S6).

No involvement in metal binding from D_8_ and E_10_ can be observed from the NMR data. The next form is [FeHL]^2+^, most probably the result of two cysteine residue deprotonations. As for **P1**, transient [FeH_2_L]^3+^ could not be detected by potentiometry. Deprotonation of the last cysteine residue likely leads to [FeL]^+^ formation, with the p*K*_a_ value being significantly reduced compared to the free ligand (p*K*_a_ = 8.75 and 10.11, respectively), suggesting the presence of this cysteine residue in the metal coordination sphere. The participation of all cysteine residues in Fe(II) binding is confirmed by the NMR spectra recorded at pH 8.47, at which [FeHL]^2+^ is dominating in the solution; however, the [FeL]^+^ is also present with over 30% of the Fe(II) bound (Figures 5b and 8b). The NMR data show the strongest perturbation on the signals related to C_21_, C_22_, C_30,_ D_29_, and H_31_. The binding mode for [FeL]^+^ is most probably the same as that for the [FeL]^3–^ form for **P1**: {COO^–^, N_im_, 3 S^–^} (Figure 7). The p*K*_a_ values of the last three forms ([FeLH_–1_], [FeLH_–2_]^−^, and [FeLH_–3_]^2–^) are in the range of 8.80–9.83 and most likely correspond to the deprotonation of three amide groups or three water molecules. For **P2**, similar to that for **P1**, we observed weaker perturbation on the D_29_ and H_31_ signals in the NMR spectra at alkaline pH 9.64, suggesting the displacement of these resides in the coordination sphere and the potential involvement of amides in Fe(II) binding. Because the coordination modes are the same for **P1** and **P2** for the complex forms [FeL]^3–^ and [FeL]^+^, respectively, metal coordination under a higher pH is most likely also the same for these two peptides. That means that the amide groups of **P2** are likely involved in Fe(II) binding, displacing the D_29_ and H_31_ residues and resulting in a {3 S^–^, 3 N^–^} binding mode for the [FeLH_–3_]^2–^ form. We cannot exclude that the p*K*_a_ value of 9.83 corresponds to the noncoordinating lysine residue deprotonation; however, the value seems to be too low for this residue and is likely attributed to amide group or water molecule deprotonation.

#### Zinc Complexes

**Figure 9:**
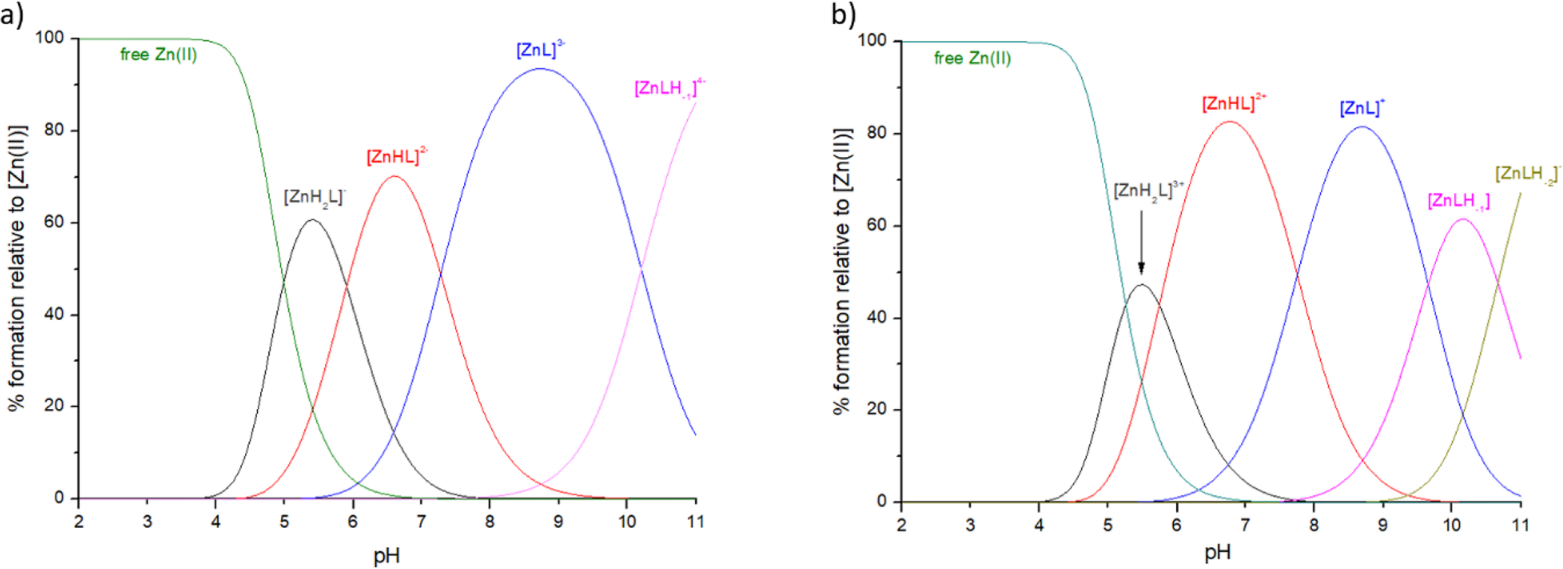
Distribution diagrams of complexes formed between Zn(II) and ligands: (a) ligand: **P1**; (b) ligand **P2**. Species distribution calculated for NMR experimental conditions: [Zn(II)]_tot_ = 1 mM and Zn(II):L = 1:1.

**Figure 10:**
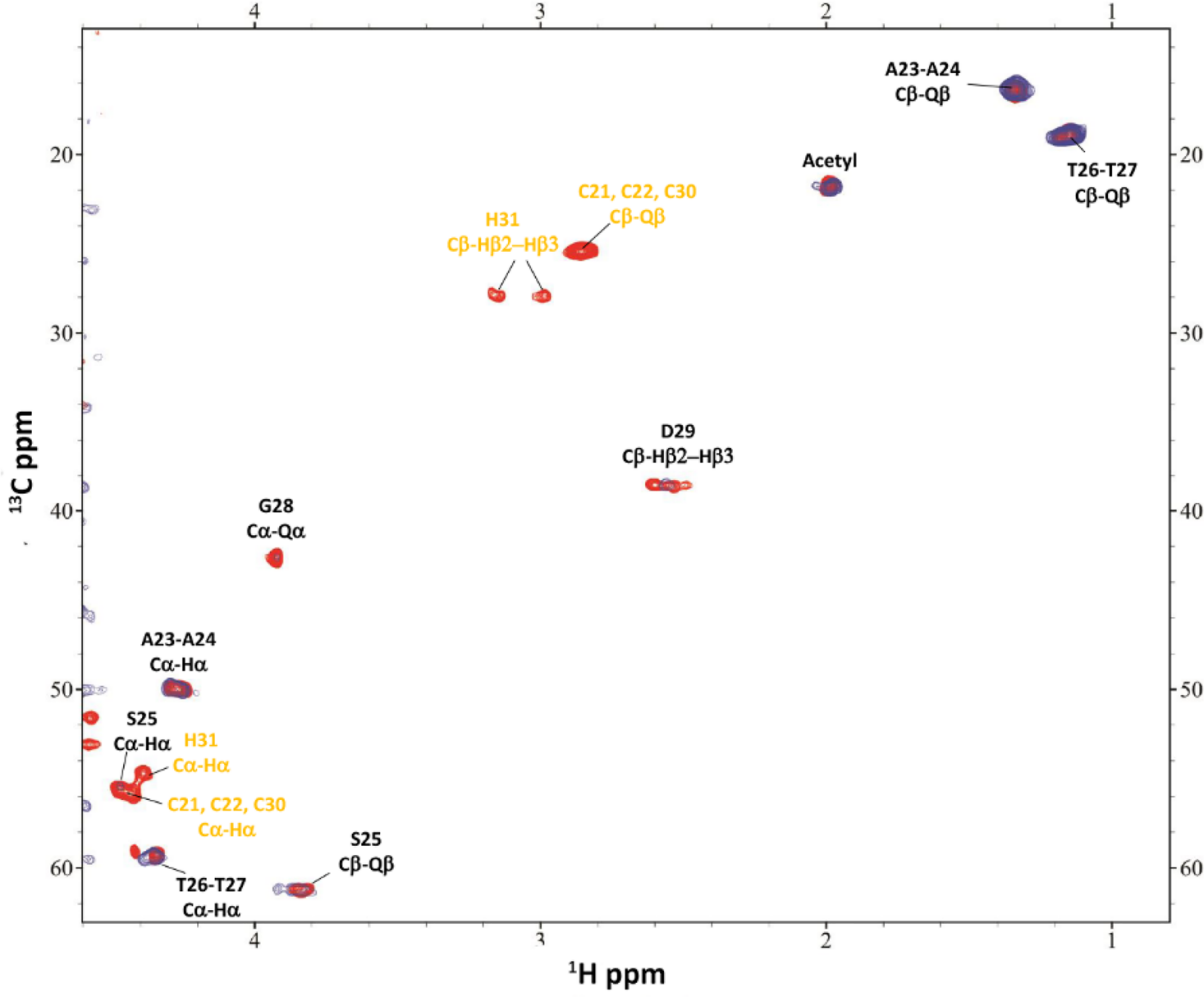
Comparison of a selection of ^1^H–^13^C HSQC spectra for the free peptide **P1** (red) and Zn(II)/**P1** system (blue) at a 1:1 molar ratio and pH 8.5.

The first Zn(II) complex with **P1** detected by potentiometry is [ZnH_2_L]^−^, which started to form at a pH of about 4 (Figure 9a). In this species, C-terminal aspartic acid (D_29_) and two cysteine residues are most probably deprotonated. The complexation of Zn(II) ions can result in a different order of deprotonation of amino acid residues compared to the free ligand titration: cysteine residues can deprotonate before histidine, which is a behavior that we have already observed for different cysteine-containing peptides.^68^ Zinc binding to **P1** causes a number of NMR resonances to exhibit fast exchange broadening upon Zn^2+^ addition. The NMR spectra recorded at pH 5.4 show the disappearance of C_20_ and C_21_ signals, confirming their participation in metal binding, in the {2 S^–^} mode (Figures S7 and S8). The signal of H_31_ is perturbed in the spectra, which could mean that it is also starting to take part in Zn(II) binding, in the form of [ZnHL]^2–^, which is already present in the solution at this pH. The NMR spectra recorded at pH 6.1, where [ZnHL]^2–^ is the main form in the solution, confirm that indeed this form results from H_31_ deprotonation and that the imidazole nitrogen atom of H_31_ is involved in the coordination of metal ion, which is consistent with the lowered p*K*_a_ value of the histidine residue in the Zn(II) complex (p*K*_a_ = 5.91) compared to the ligand (p*K*_a_ = 6.96) (Figure S8). The last cysteine residue (C_30_) deprotonation leads to the [ZnL]^3–^ complex form, including over 90% Zn(II) ions in the solution at a maximum concentration of the form at a pH of about 8.7 for **P1**. The disappearance of C_30_ signals can be observed in the NMR spectra recorded at pH 8.5; thus, C_30_ completes the tetrahedral, four-coordination binding mode of Zn(II): {3 S^–^, N_im_} (Figures 10 and S9). Perturbation of the D_29_ signal seen at this pH is most probably a consequence of its proximity to the metal-binding residues. The last deprotonation leading to the complex form [ZnLH_–1_]^4–^ probably corresponds to deprotonation of the water molecule bound to the central Zn(II) ion.

In the case of **P2**, additional acidic groups (D_8_ and E_10_) do not result in the formation of additional complex forms at an acidic pH detected by potentiometry (Figure 9b). Similarly, as with **P1**, the first complex form is [ZnH_2_L]^3+^, starting to form at a pH of about 4, with a C-terminal group and two aspartic acid, a glutamic acid, and two cysteine residues probably deprotonated. The NMR spectra recorded at a pH of 5.5 show perturbation of the C_20_, C_21_, and H_31_ residues. At this pH, two forms exist in equilibrium in the solution: [ZnH_2_L]^3+^ and [ZnHL]^2+^. That is probably why the H_31_ signal can be seen perturbed in the spectra: its deprotonation leads to [ZnHL]^2+^ formation and Zn(II) binding in the {2 S^–^, N_im_} mode, which is consistent with the results for **P1** (Figure S10). The deprotonation of C_30_ results in the [ZnL]^+^ form and completion of the metal-binding site, {3 S^–^, N_im_}, as confirmed by the NMR spectra at pH 9.3 (Figure S11). The last two forms, [ZnLH_–1_] and [ZnLH_–2_]^−^, are probably species with deprotonated water molecules. The last p*K*_a_ value (10.57) could also be attributed to deprotonation of the noncoordinating lysine residue. The stability constants of Zn(II)/peptide systems are collected in Table 3.

**Table 3:** Stability Constants (log β) and p*K*_a_ Values of the Zn(II)/Peptide Systems^a^

peptide	species	log β^b^	p*K*_a_^c^	deprotonating residue
**P1**	[ZnH_2_L]^−^	26.97(2)		
	[ZnHL]^2–^	21.06(2)	5.91	His
	[ZnL]^3–^	13.79(4)	7.27	Cys
	[ZnLH_–1_]^4–^	3.58(4)	10.21	O_water_
**P2**	[ZnH_2_L]^3+^	26.65(4)		
	[ZnHL]^2+^	20.91(2)	5.74	His
	[ZnL]^+^	13.16(6)	7.75	Cys
	[ZnLH_–1_]	3.51(6)	9.65	O_water_
	[ZnLH_–2_]^−^	–7.16(6)	10.57	O_water_/Lys

a *T* = 298 K; *I* = 0.1 M NaClO_4_; standard deviations are given in parentheses.

b Overall stability constants (β) expressed by the equation β([ZnH_*n*_L](^*n*+2^)^+^) = [[ZnH_*n*_L]^(*n*+2)+^]/[Zn(II)][[L]^*n*+^][H^+^]^*n*^.

c Acid dissociation constants (p*K*_a_) expressed as p*K*_a_ = log β([ZnH_*n*_L]^(*n*+2)+^) – log β([ZnH_*n*–1_L]^(*n*+1)+^).

#### Manganese Complexes

**Figure 11:**
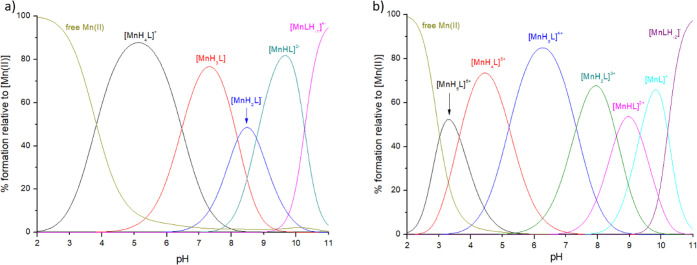
Distribution diagrams of complexes formed between Mn(II) and ligands: (a) ligand **P1**; (b) ligand **P2**. Species distribution calculated for NMR experimental conditions: [Mn(II)]_tot_ = 1 mM; Mn(II):L = 1:50.

For **P1**, the first Mn(II) complex identified in solution by potentiometric titrations is [MnH_4_L]^+^, in which the C-terminal carboxyl group and aspartic acid (D_29_) side-chain carboxyl group are most probably already deprotonated (Figure 11a). It is present in the solution already at the start of the titration and dominates from a pH of about 3.9 all the way up to 6.46. The NMR spectra recorded at pH 5.0 show the disappearance of the signals related to D_29_ and all of the H_31_ protons (Figure S12). Histidine is the C-terminal amino acid, so deprotonation can take place at the C-terminal carboxyl group and imidazole ring nitrogen. We believe that the disappearance of all of the H_31_ signals means two things: first, at pH 5.0, in the [MnH_4_L]^+^ form, the deprotonated C-terminal group binds the Mn(II) ion along with D_29_; second, imidazole nitrogen deprotonation and metal binding can already be observed at this pH because the next form, [MnH_3_L], in which histidine’s imidazole nitrogen is deprotonated, starts to form at a pH of about 4. Therefore, the metal-binding mode in the latter complex is most likely {2 COO^–^, N_im_} (Figure S13).

**Figure 12:**
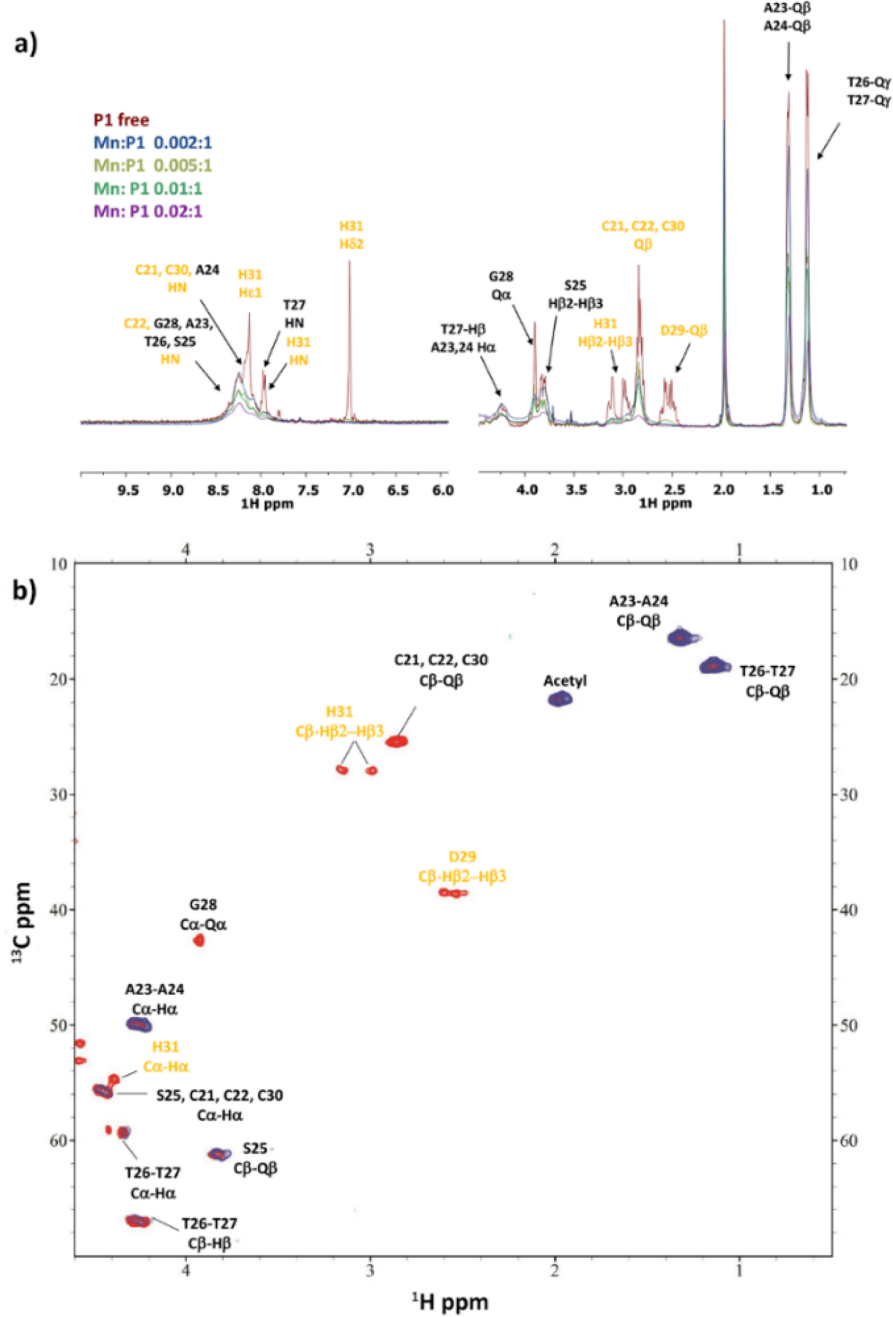
(a) Comparison of the ^1^H spectra of **P1** with increasing addition of Mn^2+^ at pH 7. (b) Comparison of a selection of ^1^H–^13^C HSQC spectra for the free peptide **P1** (red) and Mn(II)/**P1** system (blue) at 0.02:1 molar ratio and pH 7.

The most probable deprotonation of subsequent cysteine residues results in the formation of [MnH_2_L]^−^ and [MnHL]^2–^ complexes. The next two dissociations leading to the [MnLH_–1_]^4–^ species correspond to deprotonation of the third cysteine residue and probably a water molecule. The [MnL]^3–^ complex form could not be detected by potentiometry because it is probably just a transient form with a very low concentration in the solution. Whether the cysteine residues are involved in metal binding is not entirely clear. The p*K*_a_ values of the cysteine residues in complexes with Mn(II) are 8.19 and 8.77 (the third p*K*_a_ value could not be calculated from potentiometric titrations), whereas in the free ligand, the values are 8.15 and 8.92, respectively. No, or very slight, lowering of the p*K*_a_ value in the complex most probably means that cysteine residues are not involved in metal binding. However, the NMR spectra recorded at pH 7.0, at which [MnH_3_L] predominates in the solution and [MnH_2_L]^−^ starts to form, show a decrease in the intensity of the overlapped cysteine signals (Figure 12). The decrease is significant and probably corresponds to more than one cysteine residue being affected by the metal’s presence. This could mean that either the signal of C_30_ disappears because of its proximity to the D_29_ and H_31_ binding residues and one of C_21_ or C_22_ binds to the metal ion or both of them are involved in Mn(II) binding, whereas C_30_ is oriented in the solution in the opposite way with respect to D_29_ and H_31_ and experiences only a scarce paramagnetic effect. Taking all of that into consideration, the binding mode of the Mn(II) ion from a pH of 7 and above could be {2 COO^–^, N_im_}, {2 COO^–^, N_im_, S^–^}, or {2 COO^–^, N_im_, 2 S^–^}.

**Figure 13:**
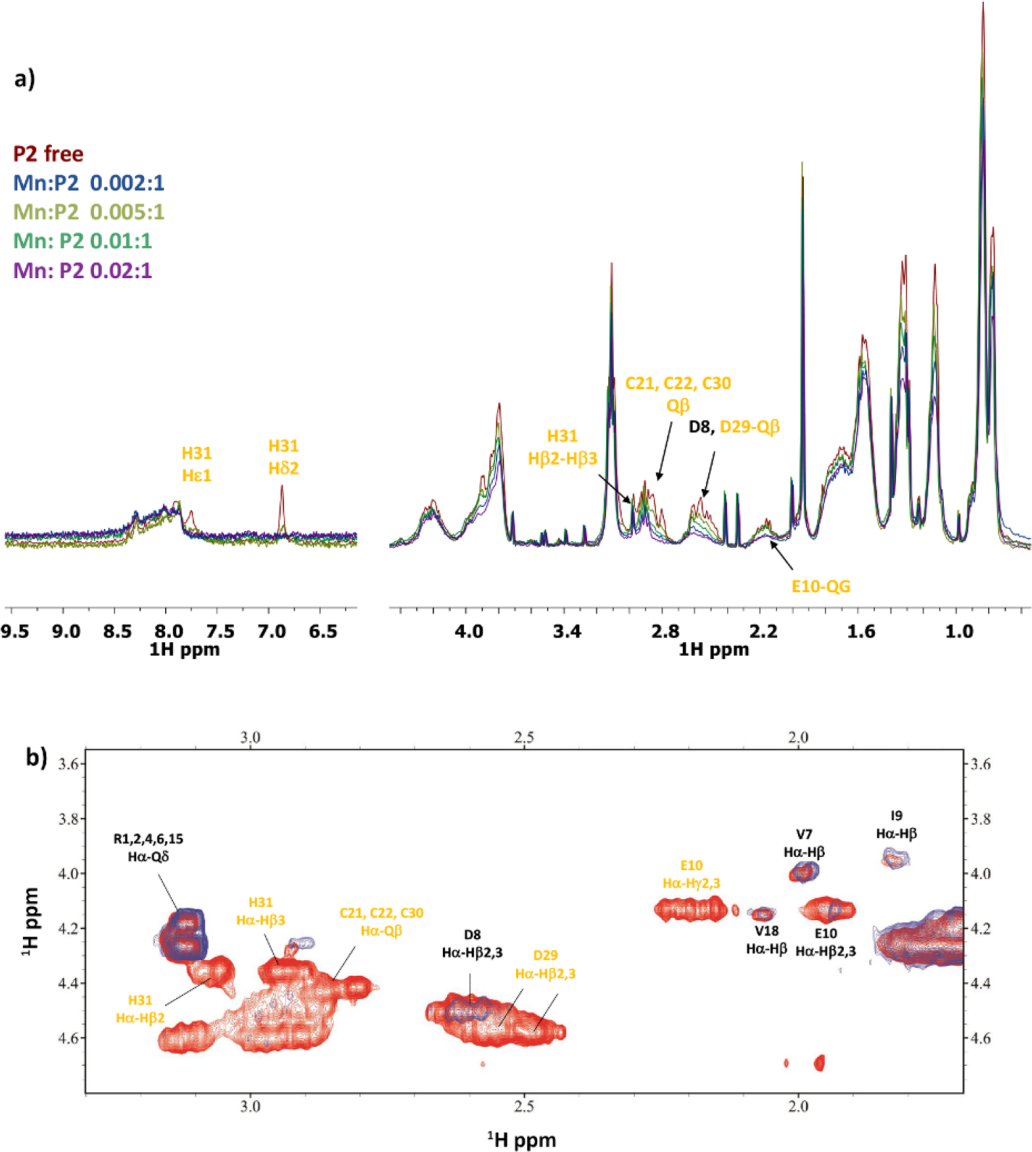
(a) Comparison of the ^1^H spectra of **P2** with increasing addition of Mn^2+^ at pH 7.4. (b) Comparison of a selection of ^1^H–^1^H TOCSY spectra for the free peptide **P2** (red) and Mn(II)/**P2** system (blue) at 0.02:1 molar ratio and pH 7.4.

In **P2** complexes, additional aspartic (D_8_) and glutamic (E_10_) acid residues compared to **P1** resulted in the detection of one more complex form under acidic pH: [MnH_5_L]^6+^, in which a C-terminal carboxyl group and two aspartic acid residues (D_8_ and D_29_) most probably exist in the deprotonated form (Figure 11b). It is present in the solution from the start of the titration and dominates in a narrow range of pH from about 3 to 3.64. Deprotonation of the possibly glutamic acid (E_10_) residue results in the [MnH_4_L]^5+^ form, with a maximum concentration at a pH of about 4.50. In the next species, [MnH_3_L]^4+^, most likely the histidine’s (H_31_) imidazole nitrogen, is deprotonated. This form binds over 80% Mn(II) ions in the solution at a maximum concentration at a pH of about 6.3. The NMR spectra recorded at a pH of 5.5, in which [MnH_3_L]^4+^ predominates, indicate metal binding by E_10_, D_29_, and H_31_. The binding mode is most probably {2 COO^–^, N_im_} or {3 COO^–^, N_im_} depending on whether the C-terminal carboxylic group of histidine (H_31_) is involved in the metal binding. The next three forms, [MnH_2_L]^3+^, [MnHL]^2+^, and [MnL]^+^, arise most possibly from deprotonation of the three cysteine residues (C_21_, C_22_, and C_30_). At a pH of 7.4, the NMR spectra show the disappearance of all cysteine signals (Figure 13). At this point, in the solution, [MnH_3_L]^4+^ coexists with [MnH_2_L]^3+^ with one cysteine residue deprotonated; however, [MnHL]^2+^ containing two deprotonated cysteine residues starts to form. Due to the paramagnetic effect of Mn(II) on the NMR spectra and the overlapping of cysteine signals, it is difficult to discern between the signals of the individual cysteines. We believe that at least one of the cysteine residues is involved in metal binding, either by directly interacting with the Mn(II) ion or by solely stabilizing the complex. This interaction is probably reflected also in the potentiometric titrations, where for **P2** the complex p*K*_a_ value of the first cysteine is lowered by 0.82 in relation to the free ligand, whereas for **P1** the p*K*_a_ value in the complex is higher than that in the free ligand. The binding modes at a pH above 7.33 could be either {2 COO^–^, N_im,_ S^–^}, {2 COO^–^, N_im,_ 2 S^–^}, {2 COO^–^, N_im,_ 3 S^–^}, {3 COO^–^, N_im,_ S^–^}, or {3 COO^–^, N_im,_ 2 S^–^}, depending on the involvement of the C-terminal group and the number of the cysteines involved in the metal binding. The last detected form is [MnLH_–2_]^−^, in which the water molecules are deprotonated (average p*K*_a_ = 10.20). Alternatively, one of these deprotonations could correspond to the noncoordinating lysine residue. The stability constants of the Mn(II)/**P1** and Mn(II)/**P2** systems are collected in Table 4.

**Table 4:** Stability Constants (log β) and p*K*_a_ Values of the Mn(II)/Peptide Systems^a^

peptide	species	log β^b^	p*K*_a_^c^	deprotonating residue
**P1**	[MnH_4_L]^+^	37.88(6)		
	[MnH_3_L]	31.42(5)	6.46	His
	[MnH_2_L]^−^	23.23(7)	8.19	Cys
	[MnHL]^2–^	14.46(7)	8.77	Cys
	[MnLH_–1_]^4–^	–6.07(4)		Cys, O_water_
**P2**	[MnH_5_L]^6+^	42.29(4)		
	[MnH_4_L]^5+^	38.65(2)	3.64	Glu
	[MnH_3_L]^4+^	33.45(3)	5.20	His
	[MnH_2_L]^3+^	26.12(4)	7.33	Cys
	[MnHL]^2+^	17.53(4)	8.59	Cys
	[MnL]^+^	8.20(4)	9.33	Cys
	[MnLH_–2_]^2–^	–12.21(4)		O_water_, O_water_/O_water_, Lys

a *T* = 298 K; *I* = 0.1 M NaClO_4_; standard deviations (3σ values) are given in parentheses.

b Overall stability constants (β) expressed by the equation β([MnH_*n*_L](^*n*+2^)^+^) = [[MnH_*n*_L]^(*n*+2)+^]/[Mn(II)][[L]^*n*+^][H^+^]^*n*^).

c Acid dissociation constants (p*K*_a_) expressed as p*K*_a_ = log β([MnH_*n*_L]^(*n*+2)+^) – log β([MnH_*n*–1_L]^(*n*+1)+^).

EPR spectra recorded for Mn(II) complexes for both ligands over a wide pH range suggest hexacoordinated metal ions in complexes of octahedral geometry. The six-line pattern is characteristic for manganese (*I*_Mn_ = ^5^/_2_), and the recorded spectra look similar to that of a Mn(II) hexaaqua ion, meaning that the geometry of the complexes is most likely octahedral, with water molecules completing the metal coordination sphere. EPR spectra for **P1** with Mn(II) are shown in Figure S14. EPR spectra for Mn(II)/**P2** are shown in Figure S15. The value of the hyperfine coupling constant (*A*) for both Mn(II)/**P1** and Mn(II)/**P2** under all measured pH conditions is about 95 G, consistent with the octahedral geometry of the Mn(II) complexes.^69,70^

### Comparison of the Thermodynamic Stabilities of Metal Complexes

Although the stability constants are a direct measure of the binding power, they cannot be used to compare compounds with various binding groups due to differences in the ligand protonation constants, which may affect the log β/log*K* values. Therefore, to compare the metal chelation efficacy of the two studied peptides between each other and with various proteins, we have used a variety of tools.

**Figure 14:**
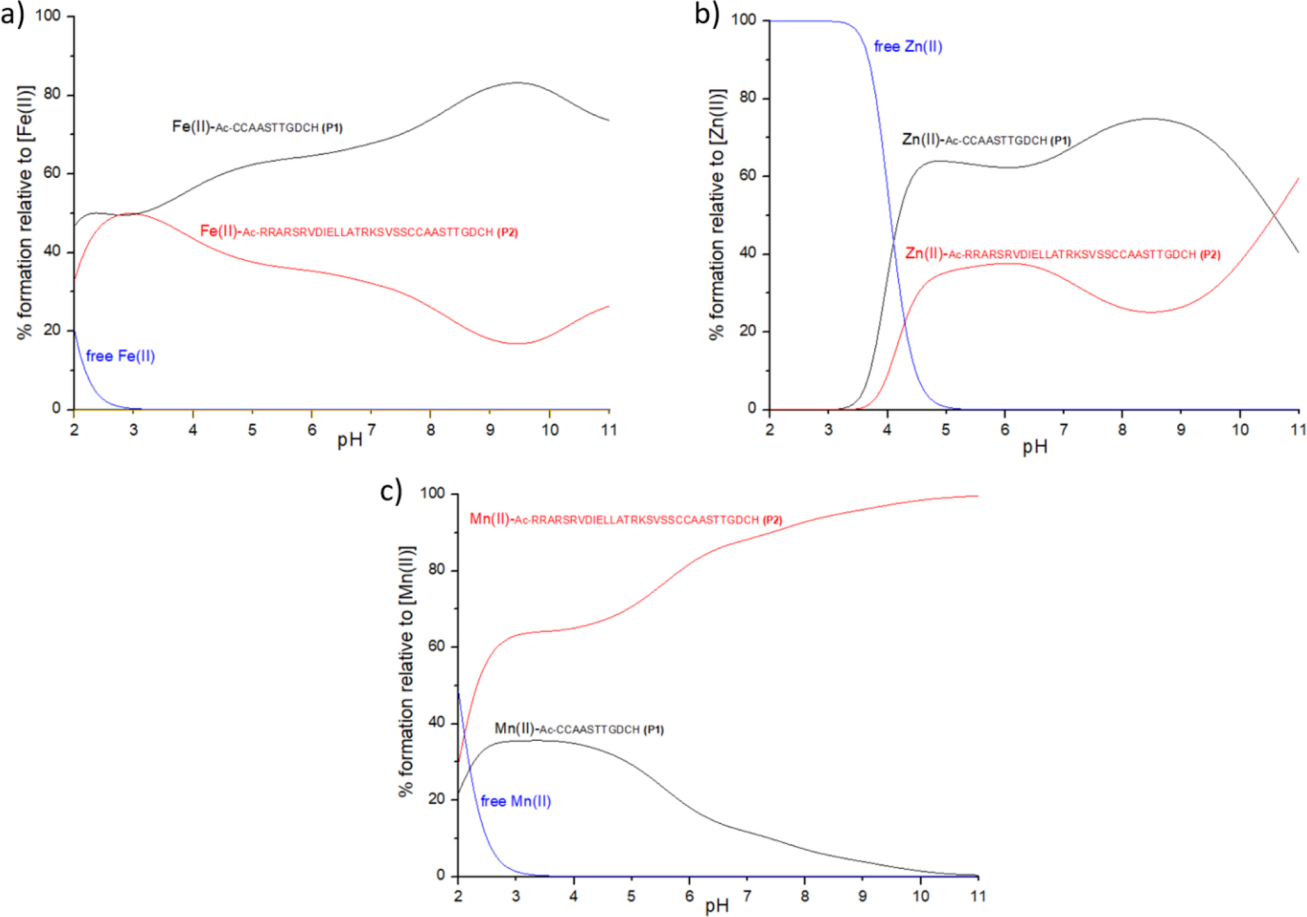
Competition plots between the ligands **P1** and **P2** and the metal ion. The plot describes complex formation in a hypothetical situation, in which equimolar amounts of all reagents are mixed. Plot a presents Fe(II) systems. Plot b presents Zn(II) systems. Plot c presents Mn(II) systems. Conditions: *T* = 298 K; *I* = 0.1 M NaClO_4_; M:L = 1:1; the concentration of all reagents is 0.2 mM.

Comparing the competition plots (a hypothetical situation in which equimolar amounts of the reagents are mixed) between the ligands and metal ion (Figure 14), a specificity can be seen for the three studied metal ions. Fe(II) and Zn(II) form stronger complexes with **P1** throughout almost the whole studied pH range, while Mn(II) forms significantly more stable complexes with **P2**, containing the whole cytoplasmic C-terminal part of *E. coli* FeoB. This behavior probably reflects the difference in the metal ion affinity for various donor groups: while Fe(II) and Zn(II) are usually considered to be borderline Lewis acids with an affinity for borderline and soft bases, such as imidazoles and thiols, Mn(II) in proteins is relatively rarely bound by cysteines and tends to interact more with harder bases, such as oxygen-based ligands (e.g., aspartates and glutamates).^44,45,71^ Our results are consistent with this behavior because **P2**, compared to **P1**, contains two more carboxyl-group-containing residues, D_8_ aspartate and E_10_ glutamate, which were shown by NMR spectroscopy to interact with the Mn(II) ion and stabilize the complex formation. The involvement of the cysteine residues in Mn(II) binding is not very clear; however, their role in complex formation does not seem to be as fundamental as the role of oxygen-based ligands, i.e., aspartic and glutamic acids, which is reflected by the clear domination of Mn(II)/**P2** over the Mn(II)/**P1** system already from pH 2.

For Fe(II) and Zn(II) systems, the behavior is contrary to that of Mn(II) because their **P1** complexes are more stable than those of **P2**. This probably reflects the stronger affinity of Fe(II) and Zn(II) for the cysteine’s sulfur atoms because elongation of the amino acid chain in **P2** did not result in the formation of stronger complexes, which means that the primary metal-binding site is most probably already present in **P1** and, as we propose, consists of three cysteine residues and histidine. While for Zn(II) we did not observe more complex forms for **P2** than **P1**, we did see one more complex form for Fe(II), similar to that for Mn(II); however, the influence of additional aspartate and glutamate is less prominent on Fe(II) complex formation and not as important as that for Mn(II). On the other hand, the weakening of the binding may be due to changes of the structure and the charge of the binding site and may be important for the physiological role of this part of the protein.

**Figure 15:**
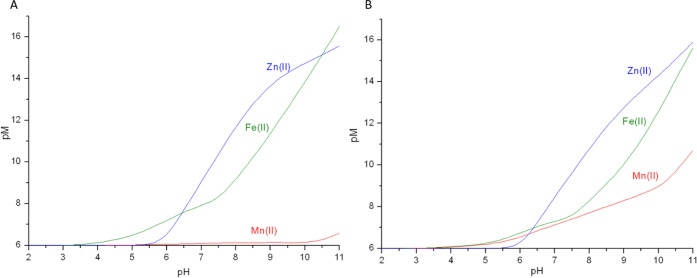
Plots of Fe(II), Zn(II), and Mn(II) pM values with the ligands: (A) **P1**; (B) **P2**. pM = −log [M]_free_ calculated for [M]_total_ = 1 × 10^–6^ M and [L]_total_= 1 × 10^–5^ M.

Because competition plots are drawn for hypothetical situations in which equimolar amounts of reagents are mixed, which is hardly ever possible in cells, we decided to utilize a more biologically relevant factor, pM, to compare the affinity of the ligands for the metal ions. The pM is defined as a negative logarithm of the free metal concentration: pM = −log [M]_free_.^72,73^ The higher the value of pM, the lower the concentration of free metal; thus, the ligand is binding the metal ion more effectively. We have calculated pM values for each system in a pH range of 2–11 and compared abilities of **P1** and **P2** to bind Fe(II), Zn(II), and Mn(II) (Figure 15). We have chosen conditions in which the concentration of metal ions is [M]_total_ = 1 μM, resembling the iron concentration in the cells and a 10-fold excess of the peptide.^74^

For Fe(II) and Zn(II), both ligands seem to be pretty equal in terms of metal binding, with **P1** being slightly more effective. For both ligands, Fe(II) is bound more strongly than Zn(II) in the acidic pH range, up to a pH of about 6.25 (pFe at this point is 7.38). **P1** binds Fe(II) more effectively than Zn(II) again above pH 10.4, most probably as a consequence of the involvement of amide groups in iron binding under strongly alkaline conditions. For **P2**, that behavior is not observed, with pZn values being slightly higher than pFe, even at pH 11. At pH 7.4, pFe and pZn values for **P1** are 8.22 and 10.12, respectively, while for **P2**, pFe = 7.51 and pZn = 9.39, consistent with **P1** being a better ligand for both of these metals than **P2**, a behavior that we have already seen in competition plots in Figure 14.

Figure 15 shows that, for Mn(II), **P2** is clearly a more efficient ligand, with pMn values being very close to those of pFe up to a pH of about 7.5, albeit never higher. For **P1**, pMn values are more or less at the level of 6 throughout the pH range, slightly rising in basic pH, although with no chance to challenge Fe(II) and Zn(II) for **P1** binding. Under acidic conditions, pMn values in the **P2** system almost match the pFe values. For example, at pH 5.5, pMn and pFe values are 6.32 and 6.44, respectively. This could mean that the whole cytosolic C-terminal fragment of *E. coli* FeoB could potentially also bind to Mn(II). At a pH of 7.4, the pMn values are 6.11 and 7.35 for **P1** and **P2**, respectively. **P2** being a definitely better ligand for Mn(II) than **P1** further highlights the divalent manganese preference for oxygen ligands (D_8_ and E_10_) present in **P2**, a behavior contrary to that observed for Fe(II) and Zn(II), which prefer more borderline sulfur (C_21_, C_22_, and C_30_) and nitrogen (N_im_ and possibly amide groups for iron) donors. Such a comparison is important input to elucidate the peptide ligand preferences and coordination chemistry of the studied metals, especially for Fe(II), for which similar literature is still lacking.

For both ligands, the stability of the metal complexes at the cytoplasm’s physiological pH 7.0 follow the Irving–Williams series, with Zn(II) complexes dominating over those of Fe(II) and Mn(II) complexes being the least stable (Figure 15).^75^ However, taking into consideration the 10^5^-fold higher concentration of Fe(II) in the cytoplasm compared to Zn(II), the C-terminal part of FeoB would most probably bind iron more efficiently than zinc in the cell.^76^

The shorter ligand, **P1**, helped us to understand the coordination chemistry characteristics of the C-terminal FeoB part. However, for a comparison of the stability of complexes obtained in this study with metal chelators operating under *in vivo* conditions, we use only the more biologically relevant **P2** (the whole cytosolic fragment of the C-terminal FeoB). In order to do that, we calculated the dissociation constant *K*_d_, which refers to the concentration of the free metal ion (expressed in molarity) when half of the ligand exists in a complex form and the other half is not complexed.^77^*K*_d_ does not depend on the ligand concentration, although it depends on the pH. Because *K*_d_ refers to the general equilibrium ML = M + L, the lower the value of the constant, the greater the stability of the complex. Under physiological conditions, plenty of endogenous Fe(II) ligands are present in the cell. Most probably, glutathione (GSH) is the dominant divalent iron ligand in cytosol (GSH concentration in cytosol: 2–10 mM), additionally providing the reducing conditions necessary to maintain iron at (+II) oxidation state.^74^ Another important ligand is citrate, present in the cell at 100 μM concentration and binding about 20% of cytosol Fe(II).^76^ Apart from the small organic ligands, there are plenty of Fe(II)-binding proteins with different functions in the cell. In the case of this study, the most relevant are (i) efficient bacterial divalent metal ion transporters, such as Fe(II)-transporting MtsA and YfeA, Zn(II)-transporting ZnuA and TroA, and Mn(II)-transporting YfeA and TroA, and (ii) metal-sensing proteins, e.g., Fur (Ferric Uptake Regulator) or MntR, regulating Mn(II) homeostasis. *K*_d_ values for these systems are collected in Table 5. It must be noted that these values have been determined at slightly different pH values, although all of them lie within the cell’s physiological pH (pH 7.0–7.5) and enable us to compare the order of magnitude of *K*_d_.

**Table 5:** Comparison of *K*_d_ Values for Studied and Biological Ligands for Fe(II), Zn(II), and Mn(II)^a^

ligand	Fe(II)	Mn(II)	Zn(II)	ref
**P2**	4.75 × 10^–7^	7.02 × 10^–7^	6.31 × 10^–8^	this work
*E. coli* Fur	1.2 × 10^–6^	2.4 × 10^–5^	1.4 × 10^–10^	(78)
*S. pyogenes* MtsA	4.3 × 10^–6^			(79)
*B. subtilis* MntR		(0.2−2) × 10^–6^		(80)
*Y. pestis* YfeA		1.78 × 10^–8^	6.6 × 10^–9^	(81)
*T. pallidum* TroA		7.1 × 10^–9^	2.25 × 10^–8^	(82)
*D. radiodurans* MntH		1.9 × 10^–4^		(83)
*Synechocystis* ZnuA			7.3 × 10^–9^	(84)

a *K*_d_ values calculated for our systems as  at pH 7.0.

*K*_*d*_ values determined for the C-terminal part of *E. coli* FeoB lie within the range of the other bacterial Fe(II), Zn(II), and Mn(II) transporters Table 5. For Fe(II), the affinity for **P2** exceeds more than 2-fold the affinity for the iron-sensing protein Fur and almost 9-fold the affinity for MtsA Fe(II) transporter. A few other reported *K*_d_ values for Fe(II) and metalloproteins are usually in the 10^–5^–10^–6^ range,^85^ being higher than the *K*_d_ value determined for **P2** (4.75 × 10^–7^). Thus, we can clearly state that the C-terminal part of FeoB binds Fe(II) efficiently and possibly can act as either (i) a region binding ferrous iron in the cytoplasm after transport through the bacterial membrane and passing the metal ion on to a higher-affinity cytoplasmic ligand and/or (ii) an iron sensor region, with an affinity toward Fe(II) relatively similar to that toward a Fur protein, which could bind intracellular ferrous iron and possibly regulate the FeoB activity. However, with scarce literature regarding *K*_d_ values for Fe(II) and endogenous ligands and the various methodology used for their determination, their comparison should be treated with caution. Additionally, the C-terminal FeoB affinity for Fe(II) is only about 1.5-fold higher than the affinity for Mn(II), which could mean that Mn(II) can also be effectively bound by the cytoplasmic region of the protein. The Mn(II) affinity for **P2** is lower than the affinity for TroA transporter but higher than the affinity for MntH transporter and manganese sensor protein MntR. The Zn(II) affinity for the C-terminal FeoB is about 2-fold lower than the affinity toward TroA and about 9-fold lower than the affinity toward ZnuA transporter. Although the C-terminal FeoB is an efficient ligand also for Zn(II), its binding is probably less biologically relevant than Fe(II) and Mn(II) binding, possessing the highest affinity to studied ligands due to the Irving–Williams series. Moreover, one has to remember that the overall structure of the protein will influence the geometry of the binding and stability of the complexes. The distortions from the ideal and preferred geometries [octahedral for Fe(II) and Mn(II) and tetrahedral for Zn(II)], created by adopting a specific conformation, may be utilized by proteins to ensure the binding of the preferred metal ion and proper metalation.^71^

## Conclusions

Efficient Fe(II) transport is crucial for bacterial virulence. In this work, we have characterized the metal-binding properties of the C-terminal part of *E. coli* FeoB. We have shown that this region of the protein is an efficient ligand for Fe(II), Mn(II), and Zn(II) and could play a part either in metal binding in the cytoplasm and facilitating the transport through the membrane or as a metal sensor in the cytoplasm. With a variety of spectroscopic, spectrometric, and potentiometric techniques, we have shown the different preferences of Fe(II) and Zn(II), which prefer binding to sulfur and nitrogen atoms of cysteine and histidine residues, and Mn(II), with a preference for oxygen atoms of aspartate and glutamate. The affinity of the studied metal ions toward the C-terminal part of *E. coli* FeoB lies within the range of other bacterial divalent metal-ion transporters. This is valuable input into the coordination chemistry of the studied metal ions, especially Fe(II), because this work is one of the first solution studies to present and discuss the thermodynamics and coordination aspects of the Fe(II)/peptide complexes studied by a variety of methods, such as potentiometry, NMR spectroscopy, mass spectrometry and molecular dynamics. Concluding the results of this work, the C-terminal part of *E. coli* FeoB is a potent Fe(II) binding region, which can possibly be involved in bacterial ferrous iron transport. Similar work is carried out in our laboratories with other FeoB domains, in order to thoroughly characterize the metal-binding properties of the whole FeoB protein. Still, to fully elucidate the function of this protein *in vivo*, high-resolution and biological studies are necessary and eagerly awaited.

## Supporting Information Available

Picture of the titration vessel before and after exposure to air, distribution diagrams for the protonation equilibria of the ligands, complementary structural models, NMR and EPR spectra, and tables regarding the hydrolysis constants and ESI-MS data (PDF)
