# Supplementary material for: Fe(II), Mn(II), and Zn(II) Binding to the C-Terminal Region of FeoB Protein: An Insight into the Coordination Chemistry and Specificity of the Escherichia coli Fe(II) Transporter
Source: Inorg Chem. 2023 Nov 1;62(45):18607–24. doi: 10.1021/acs.inorgchem.3c02910 (PMC10647171; doi:10.1021/acs.inorgchem.3c02910)
Supplement: Supplementary file 1 — ic3c02910_si_001.pdf [file ic3c02910_si_001.pdf]

Supporting information for:

Fe(II), Mn(II), and Zn(II) Binding to the C-Terminal Region of FeoB Protein: An Insight into the Coordination Chemistry and Specificity of the *E. coli* Fe(II) Transporter

Bartosz Orzel, Alessio Pelucelli, Malgorzata Ostrowska, Slawomir Potocki, Henryk Kozlowski, Massimiliano Peana, and Elzbieta Gumienna-Kontecka*

Bartosz Orzel − Faculty of Chemistry, University of Wrocław, 50-383 Wrocław, Poland; https://orcid.org/0000-0001-9299-1697

Alessio Pelucelli - Department of Chemical, Physical, Mathematical and Natural Sciences, University of Sassari, 07100 Sassari, Italy; https://orcid.org/0000-0002-9502-7585

Malgorzata Ostrowska - Faculty of Chemistry, University of Wrocław, 50-383 Wrocław, Poland; https://orcid.org/0000-0001-9765-8914

Henryk Kozlowski - Faculty of Chemistry, University of Wrocław, 50-383 Wrocław, Poland; Department of Health Sciences, University of Opole, Katowicka 68, 45-060 Opole, Poland; orcid.org/0000-0001-5769-8583

Slawomir Potocki - Faculty of Chemistry, University of Wrocław, 50-383 Wrocław, Poland; https://orcid.org/0000-0002-5528-7200

Massimiliano Peana - Department of Chemical, Physical, Mathematical and Natural Sciences, University of Sassari, 07100 Sassari, Italy; https://orcid.org/0000-0002-3306-0419

Elzbieta Gumienna-Kontecka − Faculty of Chemistry, University of Wrocław, 50-383 Wrocław, Poland; orcid.org/0000-0002-9556-6378; Email: elzbieta.gumienna-kontecka@uwr.edu.pl

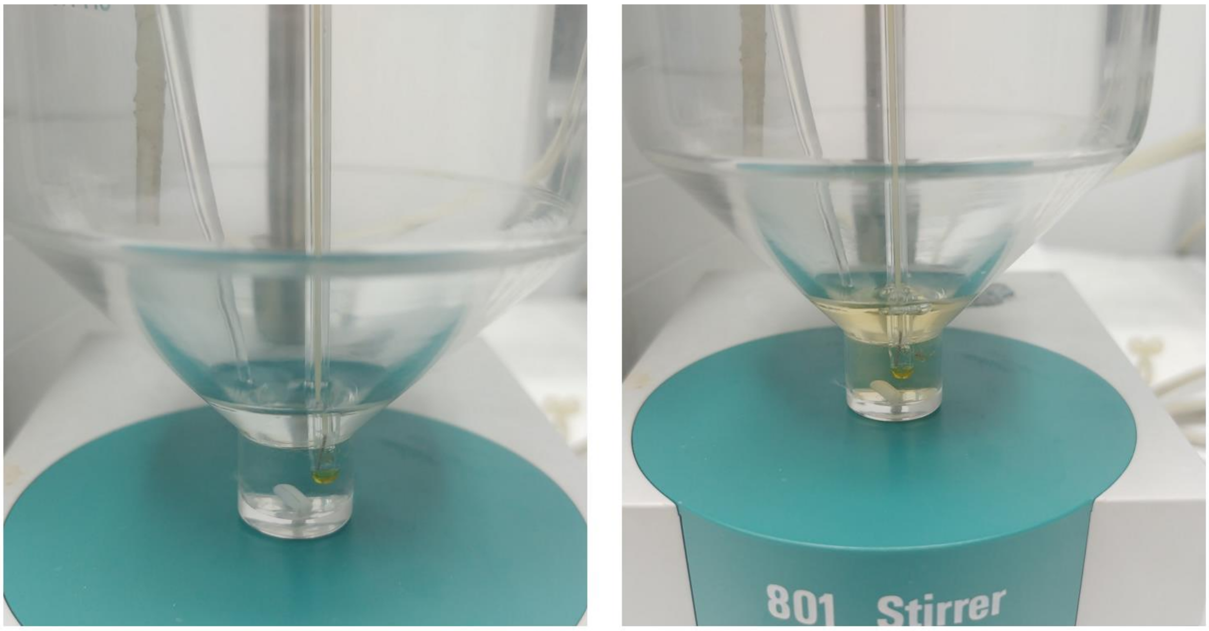

Figure S1. Comparison of the sample solution color after finished potentiometric titrations at pH=11. Left picture shows the sample in titration vessel after the finished titration- no color is observed. Right picture shows the sample 30 seconds after opening the vessel to air - yellow color indicates Fe(III) presence.

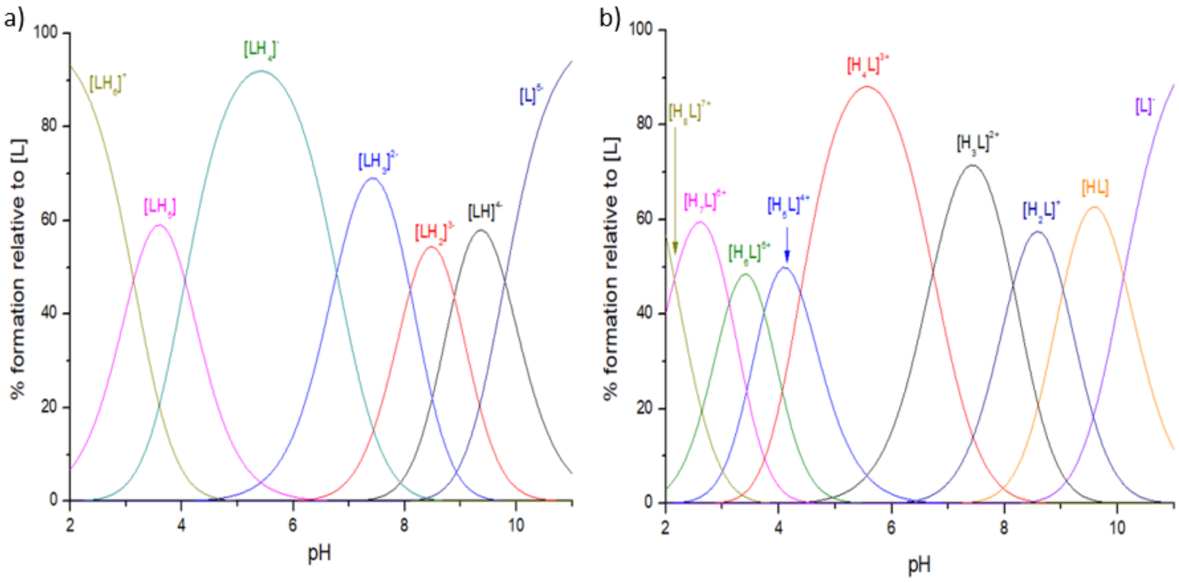

Figure S2. Representative distribution diagram for the protonation equilibria of: a) Ac_{763}CCAASTTGDCH_{773} (**P1**); b) Ac_{743}RRARSRVDIELLATRKSVSSCCAASTTGDCH_{773} (**P2**) at T=298 K and *I*=0.1 M $NaClO_4$.

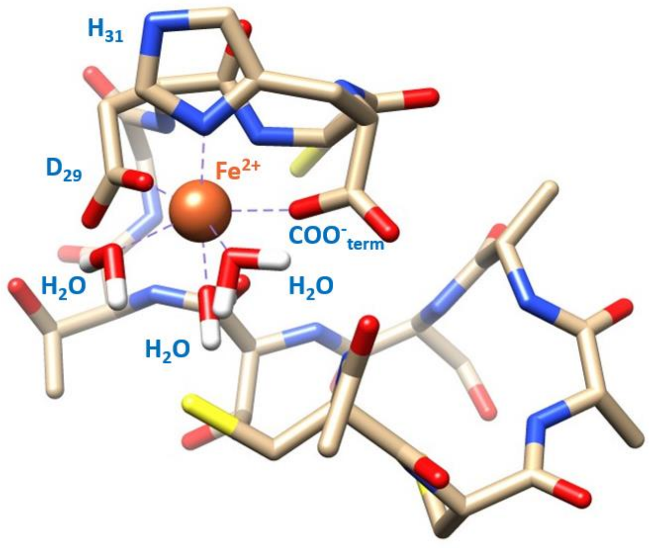

Figure S3. Structural model of the [FeH_3L], species in a {2COO^-, N_{im}, 3H_2O} coordination mode.

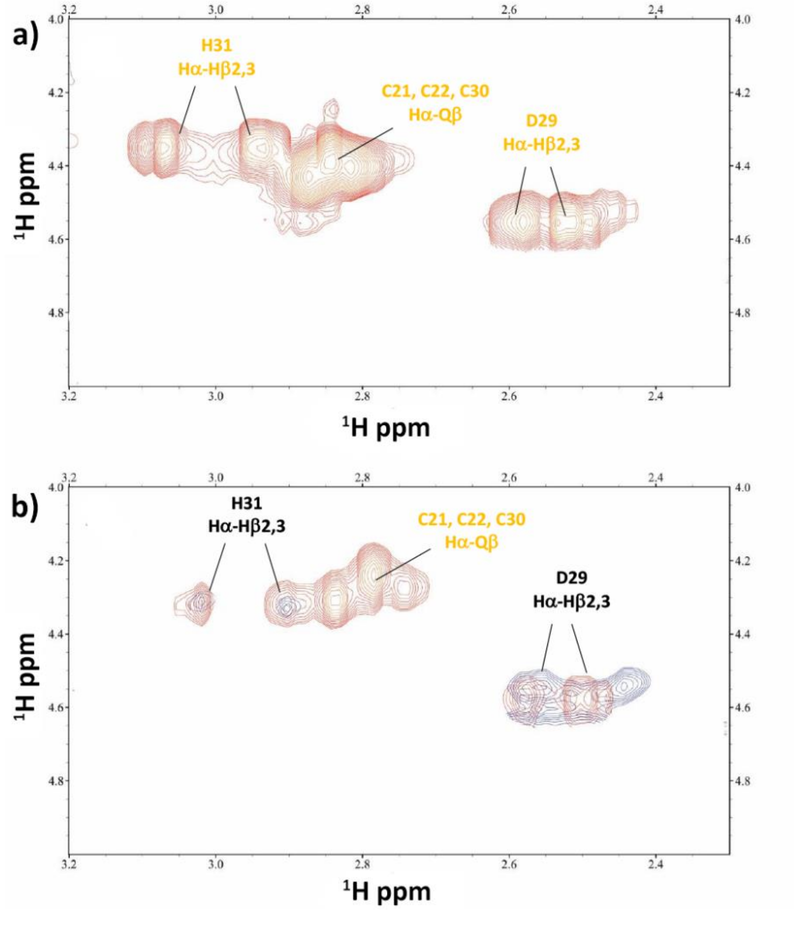

Figure S4. Comparison of a selection of ^1H-^1H TOCSY spectra for the free peptide P1 (red) and Fe(II):P1 system (blue) at 1:3 molar ratio, at pH 8.16 (a) and 9.45 (b).

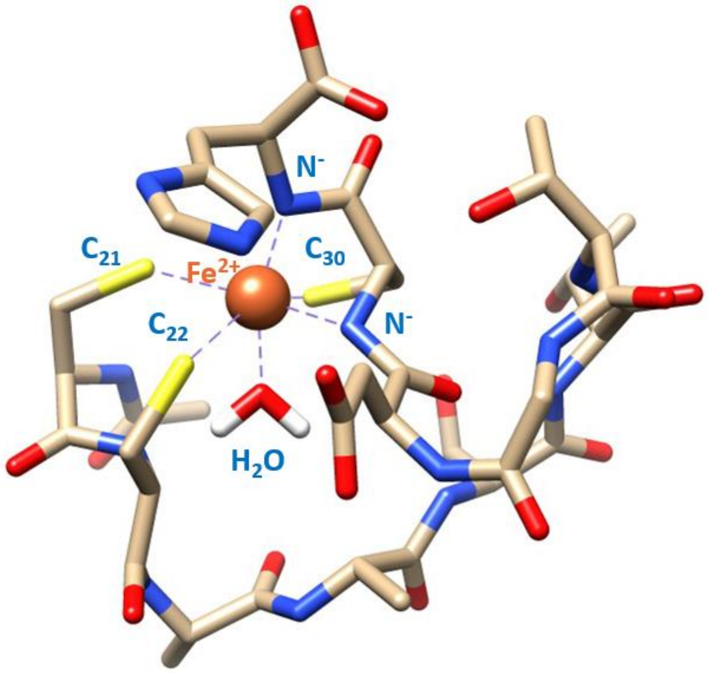

Figure S5. A proposed structural model of the $[FeLH_{-2}]^{5-}$ species in a $\{3S^-, 2N^-, H_2O\}$ coordination mode.

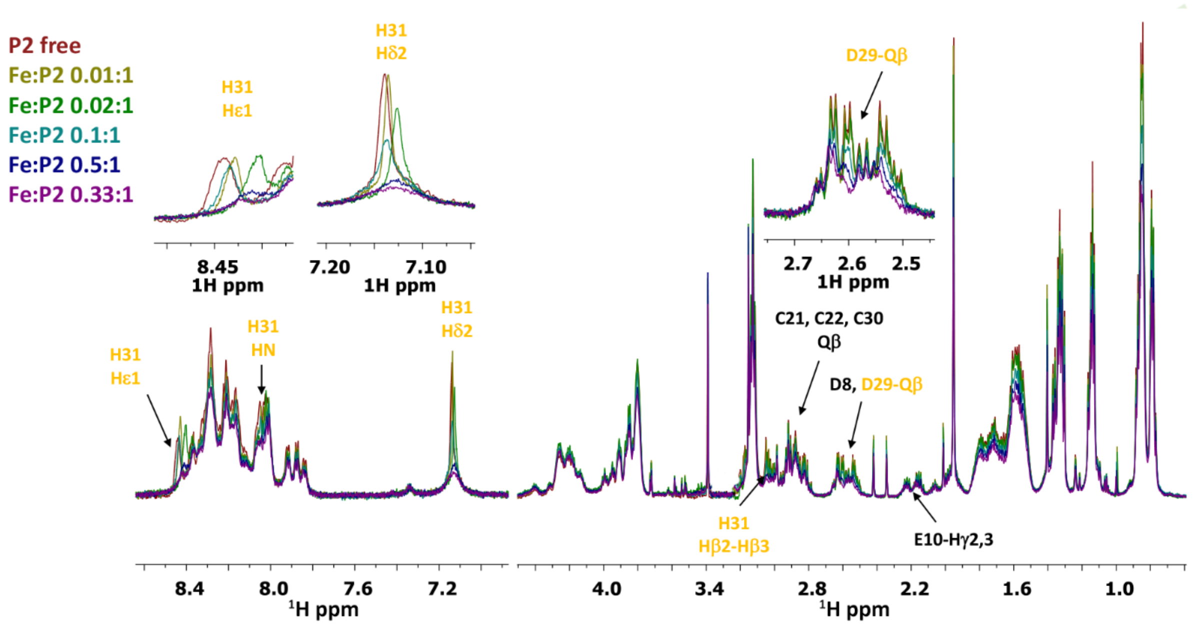

Figure S6. Comparison of ^{1}H spectra of P2 with increasing addition of Fe(II) at pH 6.2.

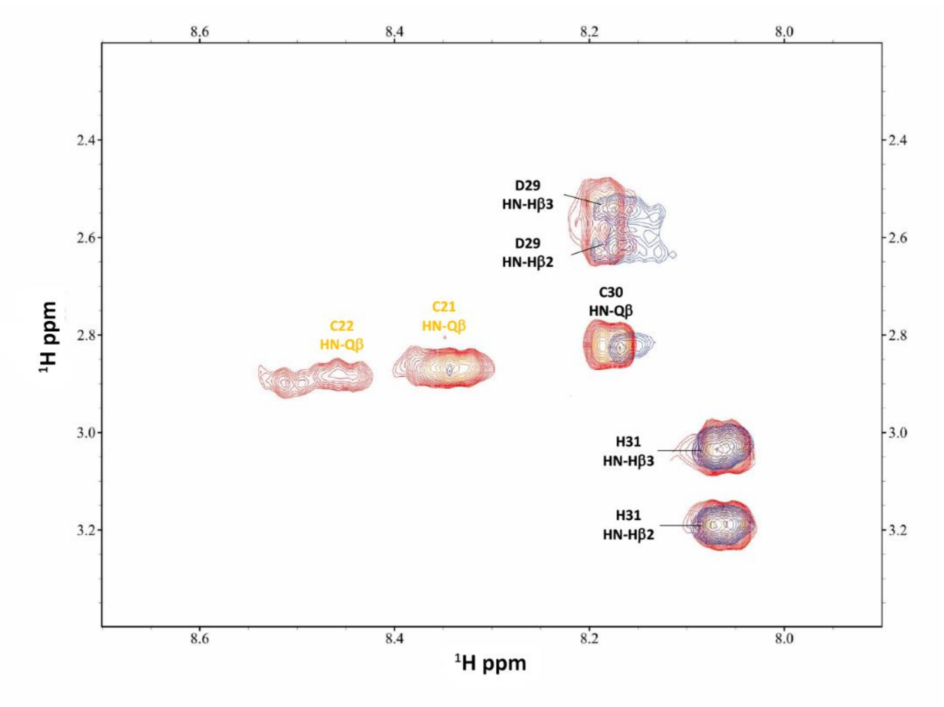

Figure S7. Comparison of a selection of ^{1}H-^{1}H TOCSY spectra for the free peptide P1 (red) and Zn(II):P1 system (blue) at 1:1 molar ratio, at pH 5.4.

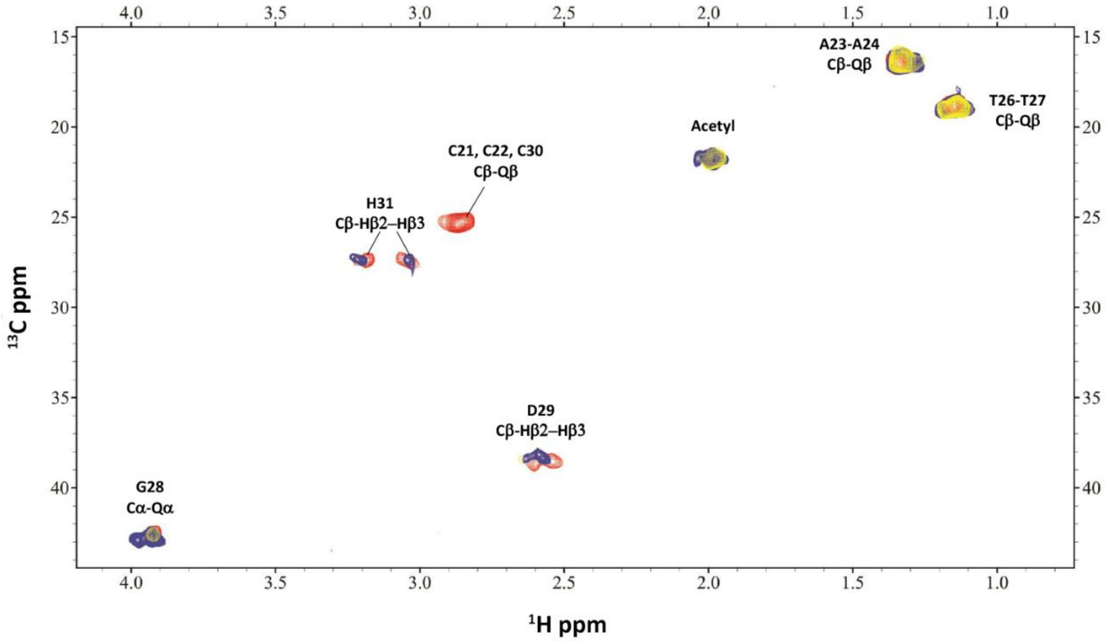

Figure S8. Comparison of a selection of ^{1}H-^{13}C HSQC spectra for the free peptide P1 (red) and Zn(II):P1 system 1:1 molar ratio, at pH 5.4 (blue) and at pH 6.1 (yellow).

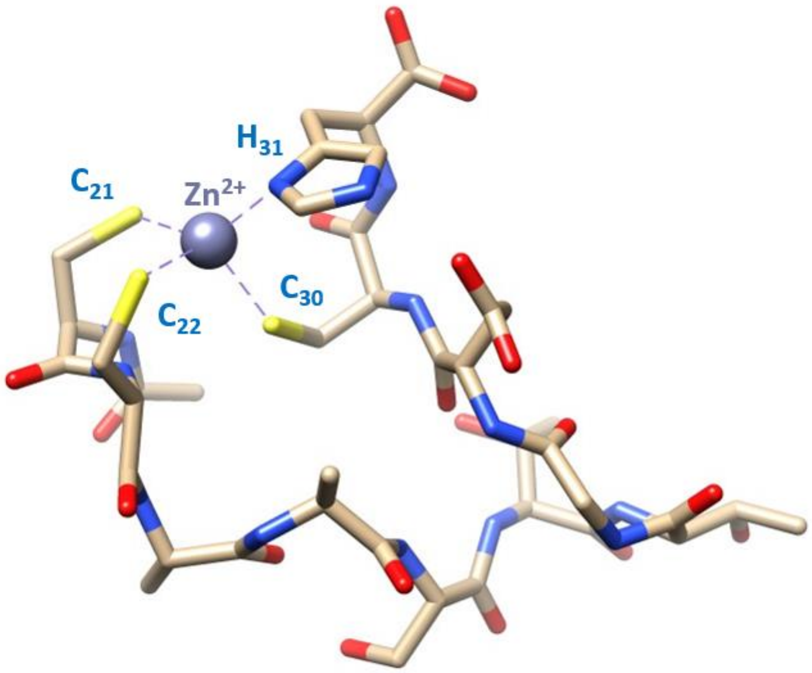

Figure S9. Structural model of the $[ZnL]^{3-}$, species in a {N_{im}, $3S^{-}$} coordination mode.

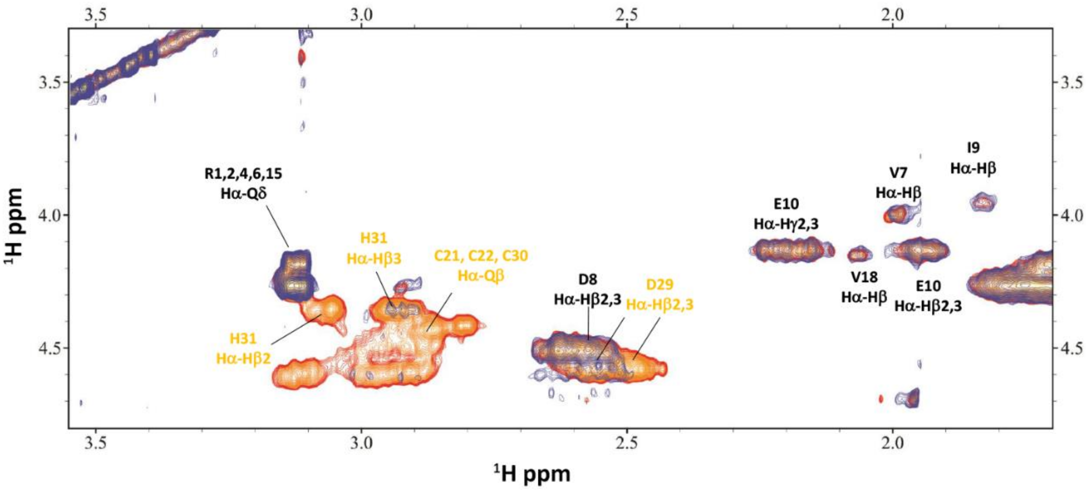

Figure S10. Comparison of a selection of ^{1}H-^{1}H TOCSY spectra for the free peptide P2 (red) and Zn(II):P2 system (blue) at 1:1 molar ratio, at pH 7.4.

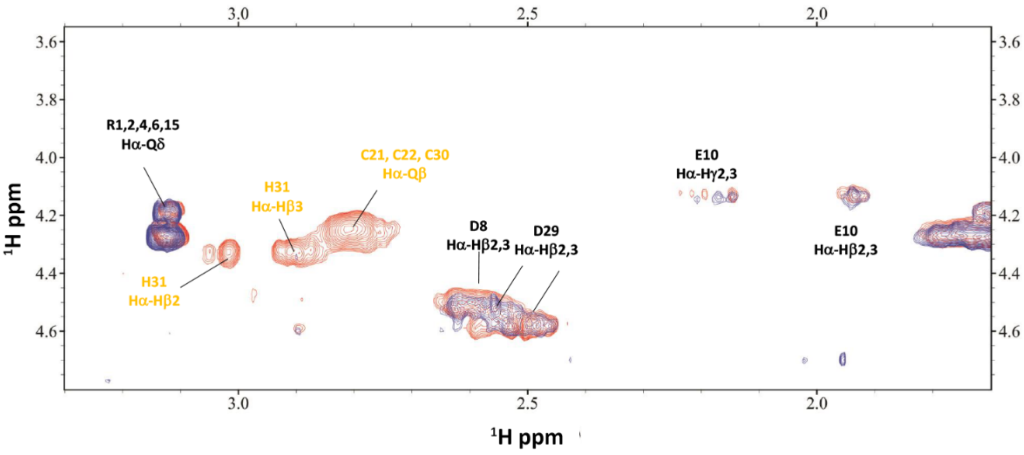

Figure S11. Comparison of a selection of ^{1}H-^{1}H TOCSY spectra for the free peptide P2 (red) and Zn(II):P2 system (blue) at 1:1 molar ratio, at pH 9.3.

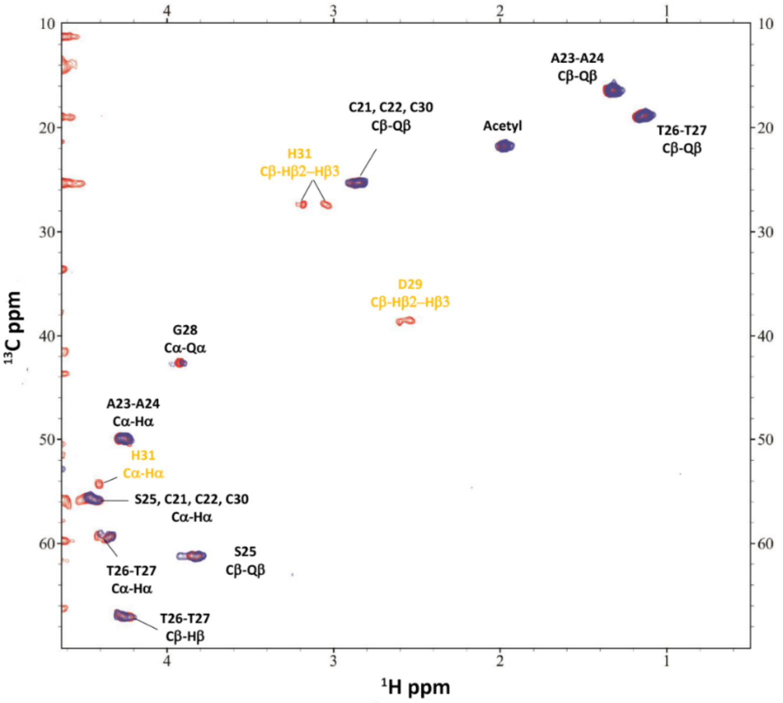

Figure S12. Comparison of a selection of ^{1}H-^{13}C HSQC spectra for the free peptide P1 (red) and Mn(II):P1 system (blue) at 0.02:1 molar ratio, at pH 5.0.

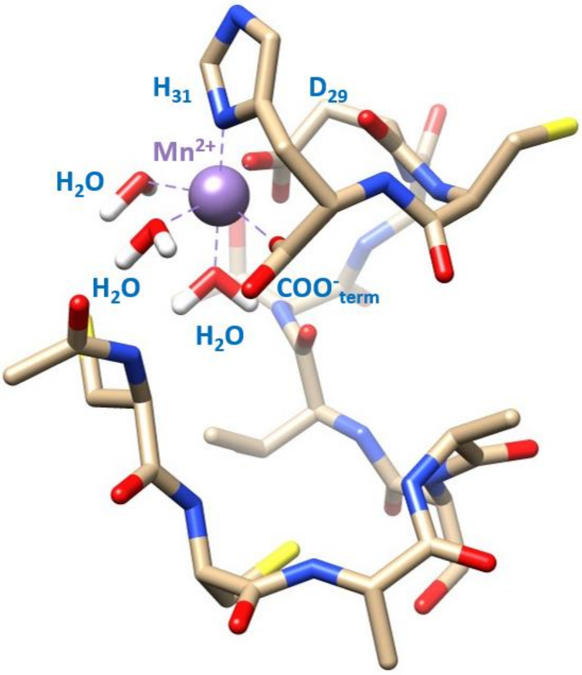

Figure S13. Structural model of the [MnH_3L] species in a {2 COO^-, N_{im}, $3H_2O$} coordination mode.

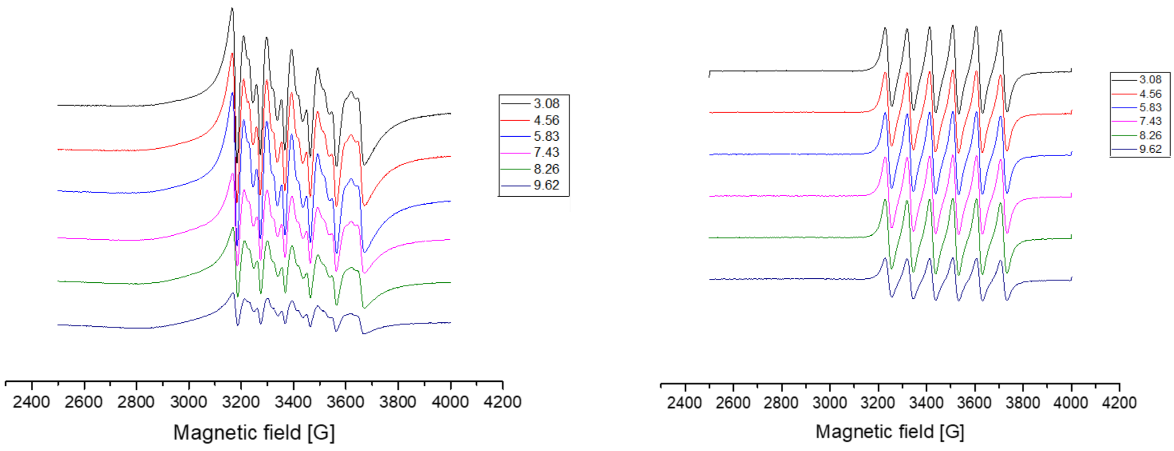

Figure S14. EPR spectra of the Mn(II):P1 system as a function of pH in: a) T= 77 K (LN); b) T= 298 K (RT).

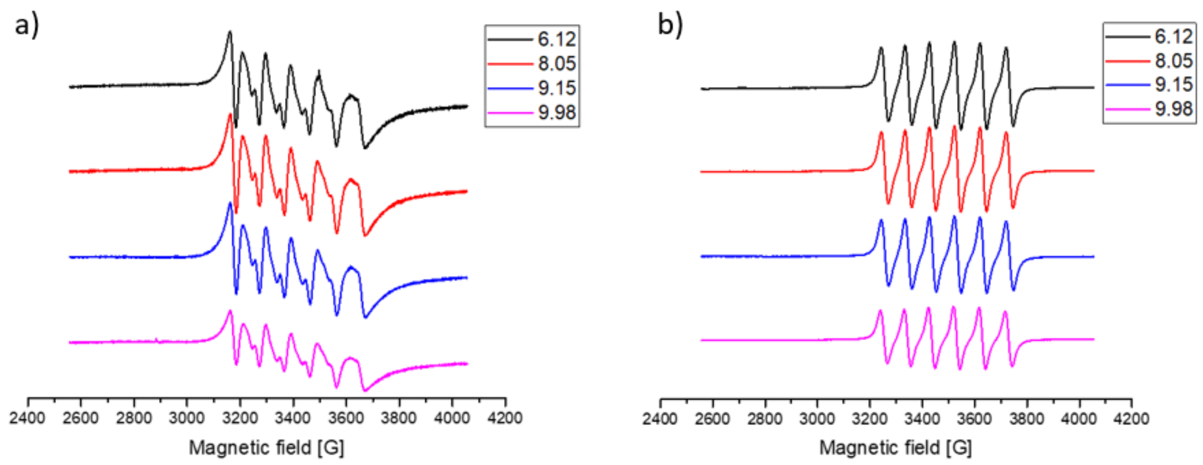

Figure S15. EPR spectra of the Mn(II):P2 system as a function of pH in: a) T= 77 K (LN); b) T= 298 K (RT).

TABLES

Table S1. Hydrolysis constants for Mn(II), Zn(II) and Fe(II) ions for I=0.1M ionic strength, T= 298 K. The hydrolysis constants for zero ionic strength were taken from the “Hydrolysis of Metal Cations” by Brown and Ekberg and calculated to 0.1 M ionic strength with the formula proposed by Baes and Mesmer in “The Hydrolysis of Cations”.[1,2]

**Species**	**log *β***
**Mn(II)**	
**$Mn(OH)^+$**	-10.78
**$Mn(OH)_2$**	-22.39
**$Mn(OH)_3^-$**	-34.34
**$Mn(OH)_4^{2-}$**	-47.82
**Zn(II)**	
**$Zn(OH)^+$**	-9.12
**$Zn(OH)_2$**	-18.08
**$Zn(OH)_3^-$**	-27.97
**$Zn(OH)_4^{2-}$**	-39.50
**Fe(II)**	
**$Fe(OH)^+$**	-9.63
**$Fe(OH)_2$**	-20.73
**$Fe(OH)_3^-$**	-32.68

(1) C. F. Baes and R. S. Mesmer: The Hydrolysis of Cations. John Wiley & Sons, New York, London, Sydney, Toronto 1976. 489 Seiten, Preis: £ 18.60. *Berichte der Bunsengesellschaft für physikalische Chemie* **1977**, *81* (2), 245–246. https://doi.org/10.1002/bbpc.19770810252.
(2) Brown, P. L.; Ekberg, C. *Hydrolysis of Metal Ions*; John Wiley & Sons, 2016.

Table S2. Intensity maxima of the major complexes and adduct ions observed by ESI-MS for each metal:peptide system at pH=7.4, M:L=1:1.

System	Ion	m/z experimental	m/z simulated
Fe(II):P1	$\{[FeH_3L]+2H^+\}^{2+}$	582.66	582.66
	$\{[H_5L]+2H^+\}^{2+}$	555.70	555.69
	$\{[H_5L]+H^+\}^{+}$	1110.40	1110.36
Zn(II):P1	$\{[ZnH_3L]+2H^+\}^{2+}$	586.65	586.64
	$\{[H_5L]+2H^+\}^{2+}$	555.70	555.69
	$\{[H_5L]+H^+\}^{+}$	1110.36	1110.36
Mn(II):P1	$\{[MnH_3L]+2H^+\}^{2+}$	582.15	582.15
	$\{[H_5L]+2H^+\}^{2+}$	555.70	555.69
	$\{[H_5L]+H^+\}^{+}$	1110.37	1110.36
Fe(II):P2	$\{[FeLH_{-1}] + 5H^+\}^{5+}$	689.93	689.92
	$\{[HL +5H^+\}^{5+}$	679.13	679.14
	$\{[HL +4H^+\}^{4+}$	848.92	848.92
	$\{[FeLH_{-1}] +3H^+\}^{3+}$	1131.22	1131.23
Zn(II):P2	$\{[ZnLH_{-1}] + 5H^+\}^{5+}$	691.53	691.53
	$\{[HL]+5H^+\}^{5+}$	679.14	679.14
	$\{[HL+4H^+\}^{4+}$	848.93	848.92
	$\{[ZnLH_{-1}] +3H^+\}^{3+}$	1151.87	1151.87
	$\{[HL]+3H^+\}^{3+}$	1131.23	1131.23
Mn(II):P2	$\{[MnLH_{-1}] + 5H^+\}^{5+}$	689.72	689.72
	$\{[HL]+5H^+\}^{5+}$	679.15	679.14
	$\{[HL]+4H^+\}^{4+}$	848.93	848.92
	$\{[MnLH_{-1}] +3H^+\}^{3+}$	1148.87	1148.87
	$\{[HL]+3H^+\}^{3+}$	1131.23	1131.23
